# Health Hazards Associated with Consumption of Roof-Collected Rainwater in Urban Areas in Emergency Situations

**DOI:** 10.3390/ijerph13101012

**Published:** 2016-10-15

**Authors:** Carol Stewart, Nick D. Kim, David M. Johnston, Mostafa Nayyerloo

**Affiliations:** 1 Joint Centre for Disaster Research, Massey University/GNS Science, P.O. Box 756, Wellington 6140, New Zealand; david.johnston@gns.cri.nz; 2 College of Health, Massey University, P.O. Box 756, Wellington 6140, New Zealand; n.kim@massey.ac.nz; 3 GNS Science, 1 Fairway Drive, Avalon, Lower Hutt 5010, New Zealand; m.nayyerloo@gns.cri.nz

**Keywords:** emergency rainwater tanks, earthquake, Wellington, health hazards, drinking-water quality, *E. coli*, lead, zinc

## Abstract

The greater Wellington region, New Zealand, is highly vulnerable to large earthquakes because it is cut by active faults. Bulk water supply pipelines cross the Wellington Fault at several different locations, and there is considerable concern about severe disruption of the provision of reticulated water supplies to households and businesses in the aftermath of a large earthquake. A number of policy initiatives have been launched encouraging householders to install rainwater tanks to increase post-disaster resilience. However, little attention has been paid to potential health hazards associated with consumption of these supplies. To assess health hazards for householders in emergency situations, six 200-litre emergency water tanks were installed at properties across the Wellington region, with five tanks being allowed to fill with roof-collected rainwater and one tank being filled with municipal tapwater as a control. Such tanks are predominantly set aside for water storage and, once filled, feature limited drawdown and recharge. Sampling from these tanks was carried out fortnightly for one year, and samples were analysed for *E. coli*, pH, conductivity, a range of major and trace elements, and organic compounds, enabling an assessment of the evolution of water chemistry in water storage tanks over time. Key findings were that the overall rate of *E. coli* detections in the rain-fed tanks was 17.7%, which is low in relation to other studies. We propose that low incidences of may be due to biocidal effects of high zinc concentrations in tanks, originating from unpainted galvanised steel roof cladding. Lead concentrations were high compared to other studies, with 69% of rain-fed tank samples exceeding the World Health Organisation’s health-based guideline of 0.01 mg/L. Further work is required to determine risks of short-term consumption of this water in emergency situations.

## 1. Introduction

**Figure 1:**
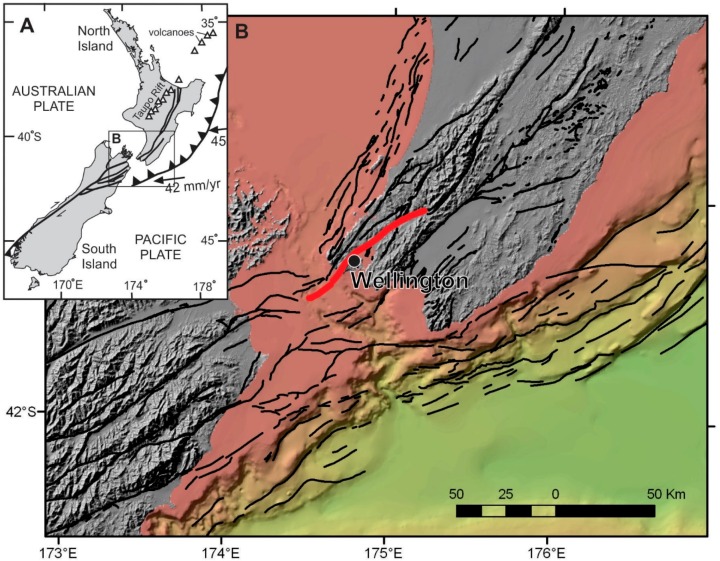
(**A**) Tectonic setting of New Zealand; (**B**) Known active faults of central New Zealand, with the Wellington Fault shown in red.

Recent studies [1,2,3] have identified that the greater Wellington region, New Zealand, is highly vulnerable to large earthquakes because it is cut by active faults, both on- and offshore (Figure 1). Wellington City is bisected by the active Wellington Fault (Figure 1), and many engineering lifelines (e.g., bulk water supply pipelines, electricity, road and telecommunications networks) cross this fault. Surface fault rupture with a large earthquake (approximately magnitude 7.5) is regarded as New Zealand’s probable maximum earthquake loss event [4,5]. The likelihood of this event happening within the next century is approximately 10% [2]. 

Bulk water supply pipelines (watermains) cross the Wellington Fault at several different locations, and there is considerable concern about severe disruption of the provision of reticulated water supplies to households and businesses in the aftermath of a large earthquake [6]. This is particularly the case for Wellington City because of its physical isolation east of the fault, its concentration of population, and the lack of access to alternative supplies. Cousins et al. [7] modelled damage to bulk watermains from a Wellington fault rupture scenario, and estimated that the minimum time to restore even a limited supply to Wellington City, following repairs, was 35–55 days (i.e., a timescale of weeks to months). Thus, there is a potential “gap” in water supply to households because water stored in reservoirs and within households is likely to be depleted within a few weeks. Recent estimates from the service provider Wellington Water suggest that, for 7 days, after a major event such as an earthquake, businesses and customers will have to be completely self-sufficient and rely on their own stored water. For days 7–30, water will be available from distribution points at a rate of 20 L per person per day, for collection by customers who may have to walk up to 1 km from their homes. From day 30 onwards, the region will move towards the restoration of normal service, but some customers may still have to collect water from distribution points. 

Attention has thus turned towards alternative measures to address this anticipated shortfall in water supplies at the household level. Hutchinson and O’Meara [8] evaluated several different options for emergency water supplies, including rainwater harvesting, surface waters, groundwater, and desalination, and concluded that rainwater harvesting showed particular promise for emergency situations. Further studies [9,10,11] have been devoted specifically to this option. 

A number of policy initiatives have been launched encouraging householders to install rainwater tanks to increase post-disaster resilience. In July 2013, the Wellington Regional Emergency Management Office (WREMO) partnered with a provider of water tanks to make inexpensive 200-litre emergency rainwater tanks available at council offices throughout the Wellington Region.

### 1.1. Microbial and Chemical Hazards in Roof-Collected Rainwater Supplies

Recent reviews [12,13,14] of the literature suggest that both microbial and chemical contamination occur in roof-collected rainwater supplies, with important sources including atmospheric deposition, leaching and weathering of roof materials, and faecal contamination. 

#### 1.1.1. Microbial Hazards

The most serious and immediate health risk associated with roof-collected drinking-water is microbial contamination. While many of the micro-organisms found in roof-collected supplies are harmless, the safety of roof-collected rainwater for human consumption will depend on excluding or minimising enteric pathogens. These include bacteria such as *Salmonella* and *Campylobacter* and protozoa such as *Giardia* and *Cryptosporidium*. These organisms are introduced by contamination with faecal material deposited by animals such as birds, frogs, lizards, rodents, possums, and insects. The microbiological quality of drinking water is commonly assessed by testing for *Escherichia* *coli* (*E. coli*) as an indicator of faecal contamination. Thermotolerant coliforms (sometimes referred to less accurately as “faecal coliforms”) are also used as indicators. 

Many studies from within New Zealand and overseas have shown that the microbiological quality of roof-collected rainwater is frequently poor. Gwenzi et al. [12], Lye et al. [13], and Ahmed et al. [15] present recent reviews of the literature. Reviews with more specific foci on Australia [16] and New Zealand [17] also include studies from the “grey” literature. For example, Sedouch [18] found that only 18% of 100 roof-collected rainwater samples from the lower North Island of New Zealand complied with the Drinking-Water Standards for New Zealand [19] (subsequently referred to as the DWSNZ), and 40 percent of samples had very high *E. coli* counts (>150 per 100 mL). Similarly, Simmons et al. [20] reported that less than half (44%) of the 125 roof-collected rainwater supplies in rural Auckland households complied with the microbiological criteria of the DWSNZ. In this latter study, specific bacterial pathogens (*Salmonella* spp.; *Legionella* spp.; *Campylobacter* spp.; and *Aeromonas* spp.) and protozoa (*Cryptosporidium* and *Giardia*) were also tested for. Of these, *Aeromonas* spp. were detected in 16% of samples and were positively associated with the presence of recent gastrointestinal disease symptoms. Eberhart-Phillips et al. [21] found that consumption of roof-collected rainwater was associated with a threefold greater risk of campylobacteriosis compared with that of non-consumers. Contamination of an open storage tank with faecal material from birds and bats at a British boarding school was identified as the most likely cause of an outbreak of *Campylobacter* gastroenteritis that affected 234 students and 23 staff [22]. While relatively few disease outbreaks have been linked to roof-collected rainwater as a source [17], this may at least partly be due to under-reporting.

#### 1.1.2. Chemical Hazards

Most chemical hazards in drinking water are of health concern only after extended exposures of years, rather than days or months, and most drinking-water guideline values for chemicals therefore relate to a level of exposure that is regarded as tolerable over a lifetime of consumption. In New Zealand, the DWSNZ prescribe maximum acceptable values (MAVs) for chemical constituents of public health significance. A MAV is the concentration of a constituent below which there is no significant risk to a consumer over a lifetime of consumption [17]. Guideline values (GVs) are also provided for chemical constituents or properties of the water that may affect the aesthetic properties of the water, such as its taste, colour, or odour, without having direct health significance.

Sources of chemical contamination in roof-collected water supplies can be divided into two types: those arising from off-site sources such as industrial emissions, vehicle emissions, and spray drift, and those arising from on-site sources, such as roof cladding, flashings, gutters, and tank materials, as well as emissions from domestic wood burners. Gwenzi et al. [12] identified several different determinants of rainwater quality, including surrounding catchment land use, leaching from roofing materials, weather patterns (especially rainfall amount and timing), and seasonal influences, such as strong winds in winter depositing marine aerosol (sea-salt spray) on roof surfaces. 

Industrial and traffic emissions are considered unlikely to cause significant impacts on the quality of rainwater collected in domestic tanks in Australia [16], and a similar situation is likely to occur in New Zealand. This is partially due to measures such as tighter controls on industrial emissions and the phasing-out of leaded petrol. In New Zealand, tetraethyllead compounds were banned as petrol additives from 1 October 1996 for health and environmental reasons [23]. Prior to these restrictions, appreciable concentrations of lead in rainfall were reported in New Zealand cities. For example, Stevenson [24] reported a mean concentration of 17 µg/L Pb in rainfall in Christchurch, and similar levels were reported in monthly rainwater samples collected in Auckland in 1982–1983 [25]. This is substantially higher than background concentrations of lead in Southern Hemisphere rainwater (0.02–0.04 µg/L), suggesting a strong urban influence [26]. 

Once rainfall lands, its quality will be affected by roof, guttering, and storage tank materials. Simmons et al. [20] in their study of 125 roof-collected water supplies in rural Auckland, reported exceedances of the NZDWS MAVs or GVs for the elements lead, zinc, copper, and arsenic. In all cases, these were attributed to inputs from system components. For lead, 14.4% of samples exceeded the MAV. Systems with either lead or galvanised iron comprising the roof, flashing, guttering, or spouting were statistically more likely to have elevated lead levels in water samples. Lower pH levels (<pH 6.5) were also associated with higher lead concentrations. Lower rates of exceedance were reported for copper (2.4% > MAV), zinc (0.8% > GV), and arsenic (14 supplies with exposed copper-chrome-arsenate treated timber components were tested for arsenic, of which one supply exceeded the MAV). A further notable feature of this study was that 74.4% of the systems sampled had alkaline pH values (pH > 7), which the authors attributed to the use of ferrocement storage tanks. Sanchez et al. [14] describe pH increases associated with storage in cement or concrete tanks as being a factor which may improve water quality within tanks, as it reduces the potential for leaching metals and is beneficial for the protection of any downstream distribution system. 

Other studies support the proposition that roof materials can influence water quality of roof-collected water supplies. Nicholson et al. [27] compared harvested rainwater quality between galvanised steel, cedar shake, asphalt shingle, treated wood, and green (vegetated) roofing materials. The treated woods yielded the highest copper concentrations (in the mg/L range), and the galvanised steel yielded the highest zinc concentrations (in the mg/L range), as compared to µg/L concentrations of these elements from the other roofing types. 

Both Mendez et al. [28] and Lee et al. [29] concluded that galvanised steel roofing was the most suitable for rainwater harvesting, compared with alternatives such as asphalt or wooden shingles, concrete or clay tiles, and green roofs, as it was associated with lower concentrations of indicator bacteria. Levels of chemical constituents generally complied with local drinking-water quality standards, particularly if systems had first-flush diverter systems in place. Concentrations in first-flush samples were typically higher than the bulk rainwater tank samples by factors of approximately two to five; for example, Lee et al. [29] reported concentrations of 428 µg/L zinc in first flush samples from a galvanised steel roof, compared with 74 µg/L in the bulk rainwater tank. 

Roofing materials have been identified as important sources of lead in rainwater tanks in a study in Brisbane, Australia [30]. Monthly samples were collected over a year-long period in 2007–2008 from 31 tanks. The Australian drinking-water guideline value for lead was exceeded by 15% of 282 samples. Source apportionment analysis indicated that factors related to the collection system contributed to 79% of the lead in the tanks on average, with “lead flashing/paint” being the dominant influence (58%). Similarly, the EnHealth review [16] identifies lead contamination as an important problem in domestic roof-collected water supplies in Australia. Of particular concern was a study by Magyar et al. [31], which reports a high incidence of lead contamination of domestic rainwater tanks in Melbourne. In pilot-scale systems, lead flashing was identified as a major source of lead. In full-scale systems, 33% of the 49 tanks in the study contained lead concentrations exceeding the recommended limit in the Australian drinking-water guidelines, by factors of up to 35.

### 1.2. Aims of This Study

In this article, we present results of a year-long investigation into the microbiological and chemical properties of six emergency rainwater tanks stationed across the Wellington Region and sampled at two-week intervals for a wide range of chemical, microbiological, and aesthetic water quality parameters. The overall aim of this exploratory study was to identify potential health hazards associated with the consumption of rainwater supplies collected for emergency use, with a further applied purpose of creating an evidence base to inform advice to residents wishing to install emergency rainwater tanks. In relation to both New Zealand-based and wider literature on water quality of roof-collected water supplies, the work presented in this report has several novel aspects. Few, if any, previous studies have addressed water quality in roof-collected water supplies for emergency use; the scientific literature is dominated by studies on rainwater harvesting for regular household supply. This may be an important distinction as property owners will be more likely to undertake regular use, cleaning, and maintenance of rainwater collection systems if the tank water is the basis of all household uses including drinking water. In contrast, an emergency tank is more likely to be installed and then neglected, and to feature limited drawdown of water. Other design features particular to the emergency water tanks used in this study are the lack of first-flush diversion systems, or features such as leaf guards intended to exclude organic debris from tanks. Furthermore, we are unaware of any previous studies that have addressed the evolution of water quality within individual tanks over time, either with respect to seasonal effects on runoff or to processes that may occur within tanks with limited drawdown.

## 2. Materials and Methods 

### 2.1. Installation of Emergency Rainwater Tanks

**Figure 2:**
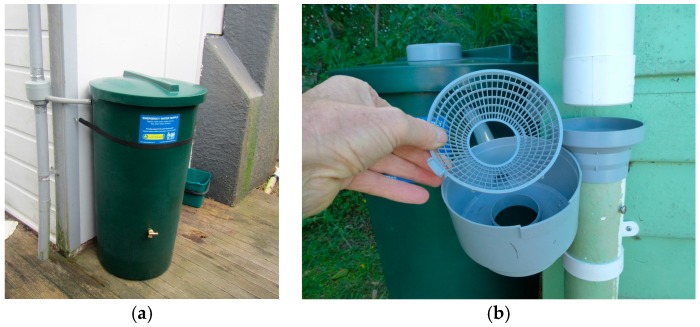
(**a**) WREMO emergency 200-L rainwater tank installed at Site 6; and (**b**) detail of diverter showing coarse screen (photo credit: Jim Cousins).

Six 200-litre emergency rainwater tanks were purchased in December 2013 and installed at properties across the Wellington region. These tanks (Figure 2) are marketed by the Wellington Regional Emergency Management office (WREMO) as inexpensive options for households wishing to increase their post-earthquake water security. 

The cylindrical tanks have a removable lid and are sold with a diverter which enables them to be connected to a downpipe. The diverter contains a coarse screen (Figure 1) to exclude large debris from the tank. The tank fills through a horizontal inlet pipe installed near the top, with inflow ceasing once the water level in the tank reaches this level. Tanks are also supplied with a brass tap and a restraining strap. The tanks are made from Rotathene^®^ 6329 linear low density polyethylene resin (LLDPE) which is resistant to ultraviolet light and compliant with both the Australian standard [32] for Plastics Materials for Food Contact Use, and the Australasian standard [33] for Testing of Products for Use in Contact with Drinking Water. 

Five of the six tanks were attached to downpipes and allowed to fill with rainwater. At Site 3, the tank was filled with Wellington town supply tapwater and not connected to a downpipe, but left as a static control. 

### 2.2. Study Sites

**Figure 3:**
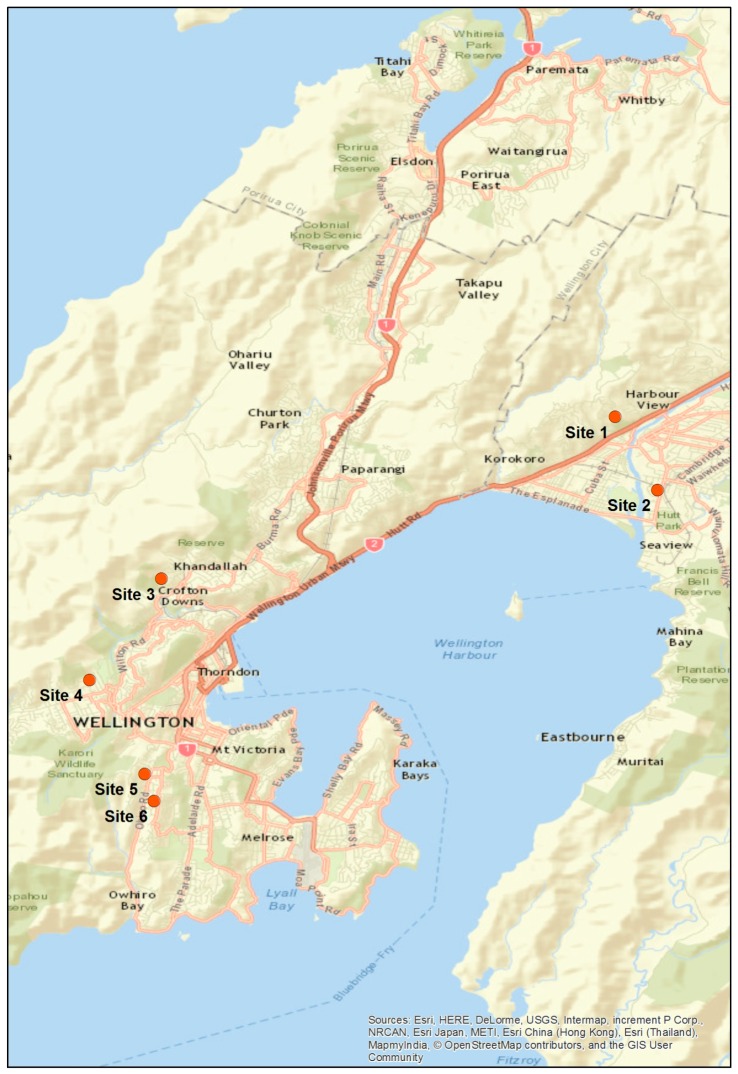
Location of study sites in Wellington region.

The six study sites are shown in Figure 3. The choice of locations was based primarily on access and availability. All sites except Site 2 (Moera) were located in hill suburbs, which are likely to be affected by stronger winds than valley floor suburbs. This may limit exposure to winter air pollution caused by domestic woodburners. All sites were located well away from major roads and thus are expected to have been relatively unaffected by contaminants associated with traffic. 

### 2.3. Corrosion Zones

All six study sites are located in Corrosion Zone C, as defined by NZS 3604:2011 [34]. This zone has a “medium” risk of corrosion to building materials via exposure to wind-driven sea-salt spray (marine aerosol). Zones are assessed by experimentally determining the mass loss rates of test panels. Zone C corresponds to mass loss of 200–400 g/m^2^/year of mild steel and 5–15 g/m^2^/year of zinc. A more recent report [35] notes that the southwest coast of Wellington is an extremely corrosive environment, with one site recording a mass loss of 692 g/m^2^/year of mild steel, and that proximity to the south coast of Wellington may cause considerable variations within Zone C. 

### 2.4. Roof Catchment Characteristics

**Figure 4:**
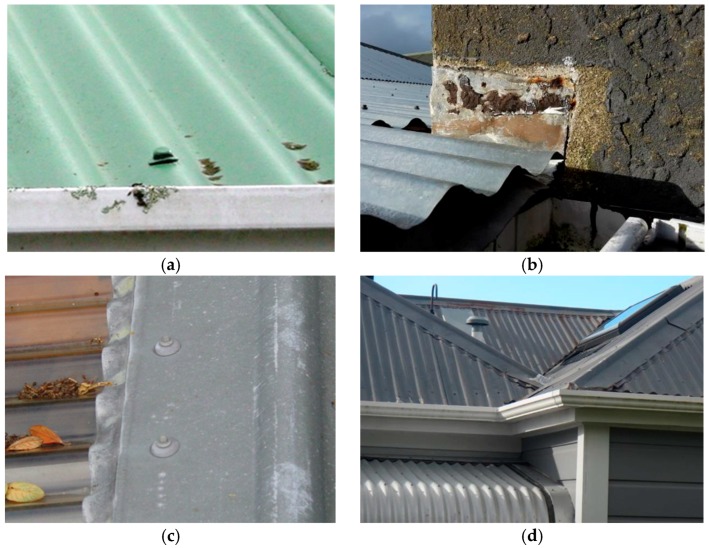
(**a**) Old lead head nail, Site 4; (**b**) Lead flashing abutting chimney, Site 6; (**c**) Soft lead edging to ridge cap, Site 1; and (**d**) Roof at Site 5, showing areas of new roof cladding at lower left (unpainted galvanised steel with modern screw fasteners) and original roof cladding on main roof (painted galvanised steel with lead fixings and flashing).

To characterise materials comprising the roof catchment systems in this study, we commissioned a survey by a registered builder experienced at building inspections. Results are summarised in Table 1 and provided in full elsewhere [36]. Specific attention was paid to the type and condition of the roof cladding, fixings (nails and screws used to fasten cladding), flashings, and guttering materials. Selected features of roof catchment systems are shown in Figure 4. Home heating arrangements for each household are also shown in Table 1. 

### 2.5. Sampling Schedule

Sampling was carried out once per fortnight for a calendar year, from 12 February 2014 to 11 February 2015. The only exception to the regular sampling was that a scheduled run on 31 December 2014 was cancelled because of the lack of availability of courier and laboratory services over this public holiday period. A further two sampling events were carried out beyond the end of the one year period, on 2 April 2015 and 1 July 2015, bringing the total number of sampling events to 28. The final two events were carried out to provide additional data on trends beyond the end of the first year.

### 2.6. Water Quality Parameters Measured

Water quality parameters measured in this study, together with their sampling frequency and rationale for inclusion, are summarised in Table 2. 

### 2.7. Sampling Procedure, Sample Storage, and Transport to Laboratory

As several different personnel assisted with sampling throughout the year, a protocol to standardise sampling procedures was drawn up. At each site, the following procedure was used:

- Recording of date and time of arrival at site (important for ensuring that samples used for indicator bacteria analysis arrived within the strict conditions imposed by the laboratory);
- Recording observations in field log;
- Flushing of tap for five seconds (to ensure that sample reflects water in main tank rather than stagnant water in tap);
- Collection of sample for indicator bacteria analysis in a sterile container using aseptic technique;
- Collection of samples for pH, conductivity and turbidity determinations in clean 250-mL polypropylene containers;
- If applicable, collection of samples for metals analysis in 250-mL plastic bottles containing nitric acid preservative;
- If applicable, collection of samples for Semi-Volatile Organic Compounds (SVOC) analysis in 500-mL amber glass containers leaving no headspace;
- If applicable, collection of samples for BTEX (Benzene, toluene, ethylbenzene, xylene) analysis in 40-mL glass containers leaving no headspace.

We note that our sampling procedures were adapted slightly as our aim was to simulate conditions under which householders will be drawing water samples from rainwater tanks in emergency situations. For example, although the commercial laboratory recommended sterilising the tap prior to collecting samples for microbiological analysis (by flaming them with a cigarette lighter), we considered it unlikely that householders will routinely do this and thus omitted it from our procedure. Similarly, we used a minimal flushing time prior to sample collection, as our assumption was that water conservation would come to the fore in an emergency situation. 

Samples were analysed both at a commercial laboratory (Hill Laboratories, Hamilton) and at Massey University, for different parameters. Samples analysed at Hill Laboratories were stored in a chilly bin and transported to the laboratory using an overnight courier service. Requirements for determining *E. coli* specify that samples must arrive within 24 h of collection, and remain at temperatures of <10 °C but above freezing point. Samples to be analysed at Massey University (Wellington) were delivered to the campus on the day of collection. When being stored for more than a few hours before analysis, samples were stored under refrigeration (on campus in a 4 °C cold room).

### 2.8. Collection of Background Rainwater Sample

A single background rainwater sample was collected on 5 November 2014 during a heavy rainfall event. The sample was collected into a glass bottle through a glass funnel. Both the funnel and the bottle were acid-rinsed and then thoroughly rinsed with Milli-Q deionised water (Milli-Q, Merck Millipore, Billerica, MA, USA). This sample was analysed using the same procedures as the tank water samples. 

### 2.9. Sample Analyses

#### 2.9.1. pH and Conductivity Determination

Analyses for pH and conductivity were carried out at Massey University (Wellington) using a pH meter (Orion 420A, Thermo Fisher Scientific Inc., Waltham, MA, USA) and a conductivity/TDS meter (HACH Model 44600, Düsseldorf, Germany). If they had been refrigerated, samples were left to warm to room temperature before analysis. Electrodes were thoroughly washed with distilled water between samples using a combination of progressively cleaner water in beakers, a wash-bottle, and dust-free tissues. 

The pH meter was calibrated against standard buffer solutions (pH 7.0 and pH 4.0) (Orion, Thermo Fisher Scientific Inc., Waltham, MA, USA) before analysis of each batch of six samples, and checked again for evidence of any instrumental drift after each batch. pH readings were allowed to stabilise before each measurement was recorded, as determined by a software function in the pH meter. Two independent (and typically very close) pH readings were taken for each sample with the reported result being their average. In cases where unusually high or low readings were recorded, the pH meter’s performance was rechecked against the pH buffers before repeating the measurement to ensure that the readings were genuine.

Conductivity measurements were collected last, after stirring the electrode through the sample solution. Accuracy of the conductivity meter was checked using a standard sodium chloride solution prepared from AR (analytical reagent) grade NaCl (Merck KGaA, Darmstadt, Germany).

#### 2.9.2. Microbiological and Chemical Analyses

Microbiological and chemical analyses (other than those detailed in Section 2.9.1 and Section 2.9.3) were carried out at Hill Laboratories, Hamilton (an IANZ-accredited laboratory). All tests reported here were performed in accordance with the terms of accreditation. Methods are described briefly below. 

Analyses for *E. coli* were carried out according to standard method 9223 B from the “Standard Methods for the Examination of Water and Wastewater” [37]. Samples were incubated at 35 °C for 24 h and a MPN (most probable number) count carried out. The detection limit of this method is 1 MPN/100 mL. 

Total concentrations of the elements Al, As, Cd, Ca, Cu, Fe, Pb, Mg, Mn, K, Na, and Zn were determined according to standard method 3125 B from the “Standard Methods for the Examination of Water and Wastewater” [38]. BTEX compounds were determined according to method USEPA 8260 B [39]. Semi-volatile organic compounds were determined according to method USEPA 8270 D [40]. 

#### 2.9.3. Analyses of Lead, Zinc, and Sodium at Massey University

To provide a more complete data set for the elements lead, zinc, and sodium, analyses were carried out on all samples using AAS at Massey University. This extended the data set from the original set of six samples collected throughout the year (Table 2) to all 28 sampling events.

For these analyses, the samples previously collected for measurement of major variables pH, conductivity, and turbidity (Section 2.9.1) were retrospectively acidified by an addition of 1800 µL 50% AR grade nitric acid (Riedel-de Haën AG, Seelze, Germany) to give a final acid concentration of 0.5%, gently shaken and left for 48 h before analysis, to reverse any adsorption that had occurred to container walls [41]. Analysis was carried out against suitably prepared matrix-matched standards on an Analytik Jena ContrAA 700 high-resolution continuum-source atomic absorption spectrometer. This dual (graphite furnace and flame) instrument features a 300 W xenon short-arc lamp operating as a continuum radiation source, a compact high-resolution double Echelle monochromator, and a CCD array detector with a resolution of approximately 1–5 pm per pixel between 200 and 800 nm [42], providing excellent background correction. Testing for lead was carried out in graphite furnace mode at a wavelength of 217.0005 nm with an ammonium phosphate matrix modifier. Zinc and sodium determinations were achieved in air/acetylene flame mode at wavelengths of 213.8570 nm and 588.9953 nm, respectively. 

The accuracy of the AAS determinations made from retrospectively acidified samples was determined by inter-laboratory comparison between the 36 samples analysed at both Hill Laboratories (using ICP-MS, or inductively coupled plasma mass spectrometry, with acidification at time of sampling) and Massey University (using AAS with samples acidified retrospectively). As can be seen from Table 3, agreement between laboratories was extremely good for sodium and zinc (mg/L level determinations), and good for lead (µg/L level determinations). We acknowledge a slight bias towards incomplete recovery in the retrospectively-acidified samples, particularly for lead. 

Overall, results of the inter-laboratory comparison confirmed the validity of incorporating AAS results for sodium, zinc, and lead to create an extended dataset for these elements.

## 3. Results

### 3.1. Summary Statistics and Relative Magnitudes

Means and standard deviations for each detected water quality parameter are provided in Table 4, as an overview of relative magnitudes across the measurement period. Full data and complete summary statistics are provided in Supplementary Materials. None of the individual trace organic compounds assayed by GC-MS were detected. A full set of all analytical data is provided in Supplementary Materials.

#### 3.1.1. pH, Conductivity and Turbidity

Mean pH levels varied between tanks, with Tank 1 being the highest (pH 6.35 ± 0.13) and Tank 2 the lowest (pH 4.74 ± 0.43). The overall mean for all rainwater tank samples was 5.56. These pH levels are low compared with other studies. Huston et al. [30] reported a mean pH level of 6.10 (range 4.2–10.2) for a large data set of 352 samples drawn from rainwater tanks in Brisbane, Australia. In a review of physicochemical and microbiological properties of roof-harvested rainwater [14], four different studies of urban rainwater harvesting systems had median pH values of 5.3, 6.2, 7.3, and 7.5. 

Mean conductivity also differed between tanks with the lowest value of 55.0 ± 13.1 µS/cm in Tank 5 and the highest value of 175 ± 64 µS/cm in Tank 6. These values are high in comparison to the limited comparative data presented in the recent review by Sanchez et al. [14], with median conductivity values of 30 and 38.2 µS/cm in two urban rainwater studies. 

Turbidity was always low; the mean value of 0.78 NTU is lower than the mean value of 1.1 NTU for 351 samples reported by Huston et al. [30]. This may reflect low particulate inputs from each roof catchment but is also in keeping with the tanks acting as depositional environments, and algal growth being inhibited by low light penetration. Where present, biological growth typically took the form of inner-surface biofilms.

#### 3.1.2. *E. coli*

In the rain-fed tanks, the overall rate of *E. coli* detections in this study was 17.7%, reducing to 12.3% if marginal detections (which are less strongly associated with health risks than clear/unambiguous detections) were excluded. This is at the low end of ranges reported in other studies (Section 1.1.1). Prevalence was markedly different between tanks, with Tanks 1, 5, and 6 reporting no *E. coli* detections throughout the entire sampling period and Tanks 2 and 4 reporting unambiguous *E. coli* in 42% and 19% of sampling events, respectively. In terms of magnitude, *E. coli* levels ranged from <1 MPN per 100 mL to 860 per 100 mL, with this high value being recorded in Tank 2 on 7 May 2014. While this level is high, it is not unprecedented. Levels of *E. coli* vary widely, as they depend on highly variable external sources (particularly bird droppings). 

#### 3.1.3. Major Elements

Elements present in mg/L concentrations were, in order of decreasing average concentration in all rainwater tanks, Na, Zn, Mg, Ca, and K. The large data set obtained for the coastal city of Brisbane, Australia [30] provides a useful basis for comparison. In the current study, average concentrations of K were approximately the same as for the Brisbane study; levels of Ca were lower; and levels of Na, Mg, and Zn were substantially higher (by factors of ~3–5). Concentrations varied markedly between tanks. 

#### 3.1.4. Trace Elements

Elements present in µg/L concentrations were, in decreasing order of mean concentration in all rainwater tanks, Pb, Cu, Al, and Mn. Iron (Fe) was not detected in any rainwater tank samples, perhaps because of its relatively high detection limit of 21 µg/L. Cadmium was not detected (<0.05 µg/L) in any of the rainwater tank samples in the first sampling run, but by the sixth sampling run was detected in four of the five tanks sampled, although at very low concentrations (maximum of 0.14 µg/L). Arsenic was consistently detected in Tank 5 only, at concentrations in the range of 3.1–5.7 µg/L. Compared with the large data set from Brisbane, Australia [30], concentrations of these trace elements were generally lower, apart from Pb, which was higher (a mean of 14.6 µg/L across all tanks compared to a mean of 5.4 µg/L for 282 rainwater tank samples from Brisbane. 

### 3.2. Evolution of Water Quality over a Sampling Period

We identify three distinct processes, each involving clusters of variables, apparently driving the evolution of water quality. These are described in the following sections.

#### 3.2.1. Accumulation Mechanism for Major Elements

**Figure 5:**
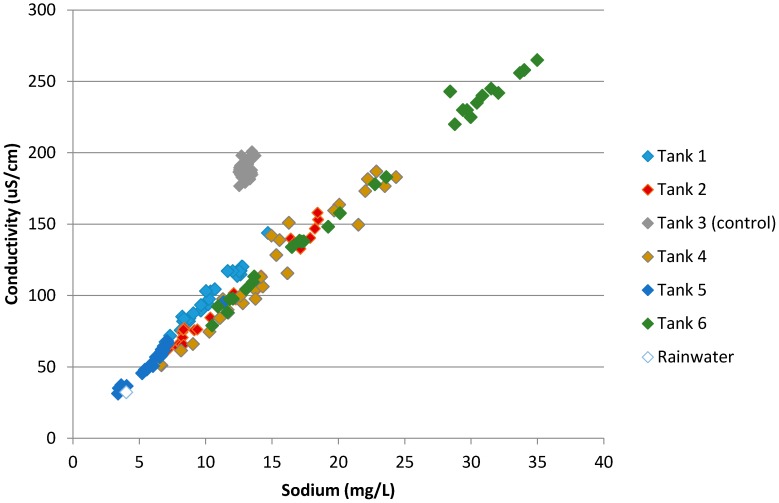
Sodium and conductivity data for all samples.

Conductivity is a measure of the total ionic strength of the water. The dominant cation contributing to conductivity in the five roof catchment tanks is sodium (Na^+^); for Tank 3 (tapwater control), the dominant cation was calcium (Ca^2+^). For further detail on a reconciliation of measured conductivities with major cation concentrations, see Table S6. Considering data from all tanks (Figure 5), a very strong positive relationship was found between conductivity and sodium for the roof catchment tanks (*r* = 0.989, *p* < 0.0001). Strong positive relationships also were found within each individual roof catchment tank (*p* < 0.0001 in all cases). 

**Figure 6:**
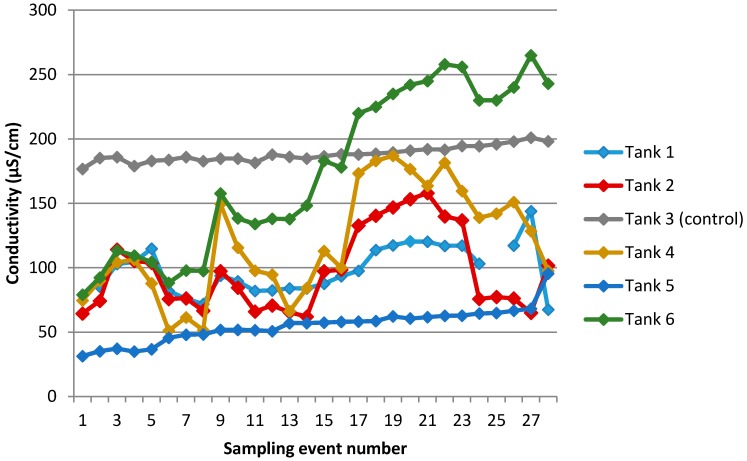
Changes in conductivity of water sampled from each tank over 28 sampling events.

Trends in conductivity over time for all six tanks are shown in Figure 6. Sampling events 1–26 are spaced at two-week intervals, but there was a seven-week interval between events 26 and 27, and a 13-week interval between events 27 and 28 (Section 2.5). For the first year of sampling (events 1–26), lines were fitted to the data. Slopes and correlation coefficients are presented in Table 5. 

Trends in accumulation differed between tanks. Tank 3 showed a minor but steady increase over time, likely to have been caused by gradual concentration through slow evaporation. Tank 5 showed a very similar trend, which is probably due to the diverter malfunctioning so that the tank was not refilled with rainwater; field notes indicated that the level slowly lowered over the duration of the experiment. 

Tanks 1, 2, 4, and 6 all had very similar ionic strength at the outset of the experiment, and remained tightly coupled for the first few sampling events, after which time they began to diverge in magnitude while retaining very similar peaks and troughs. Tank 6 showed the greatest rate of change (Table 5) and accumulated the highest conductivity (Table 4 and Table 5) and sodium concentration (Table 4). The peaks in conductivity reached between sampling events 17–22 were progressively lower for Tank 4, then Tank 2, and then Tank 1. We note that this order of accumulation (Tank 6 > Tank 4 > Tank 2 > Tank 1) corresponds to increasing distance of these sites from Wellington’s south coast (Figure 3), thus the decreasing influence of windblown marine aerosol. For further detail on source apportionment calculations to determine the contribution of marine aerosol at each site (see Supplementary Materials Section 3). 

We propose that observed conductivity trends over time in Tanks 1, 2, 4 and 6 can be explained assuming the existence of two factors: (a) a log-normal source profile (the dominant pattern for most environmental sources) and (b) a restriction on the rate of water-exchange during each rainfall event, as occurs in a water storage tank. In combination, these factors can give the strong effect of increasing in-tank concentrations over the first few months for conservative ions (those which are unlikely to be influenced by adsorption losses). This effect can occur because (with a log-normal source) at the outset of sampling, it is statistically most likely that the tank will receive low-to-moderate concentration runoff, setting a low baseline for the newly filled tank. As time goes on, however, progressively more of the infrequent but higher-concentration events will be encountered: each of these introduces a significant upward step-jump in tank water concentrations, which takes time to partially reverse with cleaner water due to the restricted water turnover. This mechanism initially causes an “upward ratchet” effect on in-tank concentrations; however, over the longer term, concentrations will oscillate around a plateau reflecting the mean of the concentration history, as seen in the results for most tanks beyond 6–8 months. In a previous study [43], we used a simple model based on assumptions of 15% water turnover per 40 mm rainfall, and a log-normal source profile for sodium, and were able to simulate trends in sodium accumulation over time.

#### 3.2.2. Microbial Indicators, pH, and the Apparent Influence of Zinc

##### Trends in Zn Concentrations

**Figure 7:**
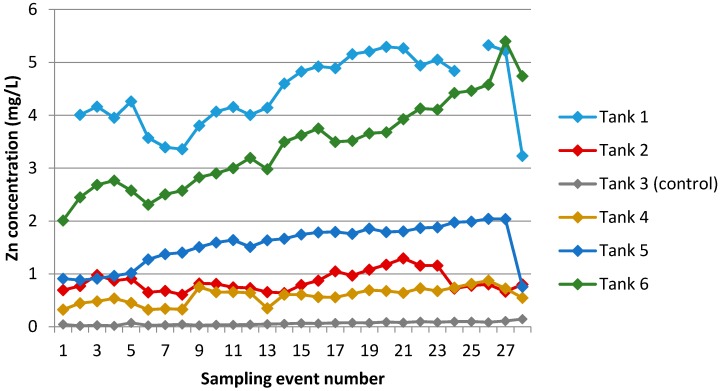
Changes in zinc concentrations in water sampled from each tank over 28 sampling events.

A time-series graph showing changes in Zn concentrations over the sampling period is provided in Figure 7. Relationships between pairs of variables (time/sampling event number, Zn and Na concentrations, pH, and conductivity) are shown in Table 6. 

At all sites with rainwater-fed tanks, roof cladding material was galvanised steel, either relatively new and unpainted (Sites 1 and 6), old and painted (Sites 2 and 4), or a combination of both (Site 5). Water in these tanks quickly took on Zn in the mg/L (part per million) range, which is approximately two orders of magnitude greater the Zn concentration in a background rainwater sample (0.027 mg/L). As for Figure 6, increases over time are apparent in Zn concentrations in some tanks (Table 6). Similar peaks around sampling events 4, 9, and 17–22 are also apparent, to varying extents, in the data for Tanks 1, 2 and 6. 

Within each roof catchment tank, highly significant positive relationships existed between concentrations of Na and Zn, and conductivity and Zn (Table 6) for all rain-fed tanks except Tank 5 (which showed similar but weaker relationships). This could indicate a common source for all these parameters, a coincident mechanism affecting all the parameters, or both. In terms of a common source, we note that Na is strongly associated with the deposition of sea-salt aerosol. Following a period of significant salt deposition, first-flush rainwater would be expected to contain high levels of sodium ($Na_{aq}^{+}$), chloride ($Cl_{aq}^{–}$), and (therefore) conductivity and is also likely to contain higher than normal concentrations of dissolved zinc, mobilised into rainfall runoff as its aqueous chloride [44]. Alternatively, or concurrently, it is also possible that zinc within tanks is subjected to the accumulation mechanism described in Section 3.2.1. 

##### Trends in pH

**Figure 8:**
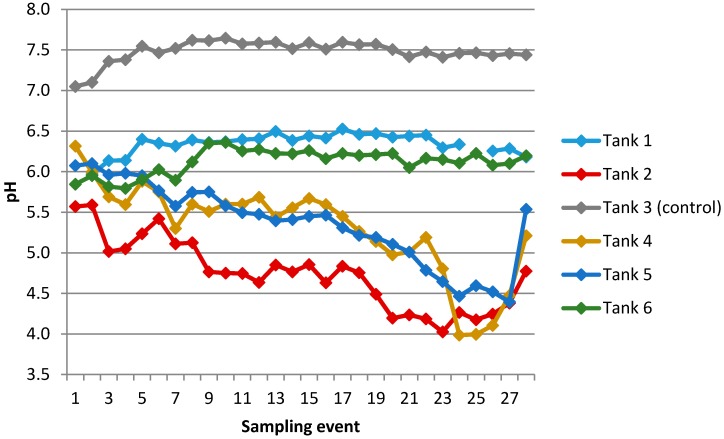
Changes in pH in water sampled from each tank over 28 sampling events.

A time-series graph showing changes in pH over the sampling period is provided in Figure 8. The progressive increases in acidity observed in Tanks 2, 4, and 5 are notable because of the unusually low pH values attained, to just under pH 4.0 in Tank 4. A further 400-L storage tank (draining a catchment comprised of polycarbonate cladding) sampled on two occasions as a supplement to this study reached an even lower pH value of 3.2. This is consistent with the generation of dissolved organic acids in these poorly buffered water systems. Fulvic acids in particular can be very acidic, with an indicative pK_a1_ value of 2.0 ± 0.3 based on four- and five-site models of carboxylate groups [45,46]. Results for non-purgeable organic carbon (NPOC) (Table 4) allowed a check to be run on this possibility by providing an upper limit on the possible concentrations of dissolved organic acids. At or near to the weeks where their lowest pH values had developed, NPOC concentrations in Tank 2 (pH 4.03) and Tank 4 (pH 3.99) were measured at 1.4 mg/L in both tanks. Based on mean molecular weights for Suwannee Stream fulvate (1150 g/mol as a number average, and 1910 g/mol as a weight average, [47]), a NPOC concentration of 1.4 mg/L would translate to molar concentrations of ~1 × 10^−6^ M, assuming all NPOC in Tanks 2 and 4 was present in a broadly similar form as the stream fulvate. Assuming a pK_a1_ value of 2.0, this concentration of fulvic acid would indeed yield a calculated pH of 4.0 (via the standard equation: pH = 0.5(pK_a_-log [HA]). 

This proposed mechanism then raises the question of how organic acids were generated from the small amounts of organic matter entering each tank. We suggest that this process is likely mediated by microbial activity. Domestic rainwater tanks may host a taxonomically diverse range of microbes, with abundances and nutrient cycling behaviour indicative of functional micro-ecosystems [48]. In a study of 80 samples from 22 tanks across Eastern Australia, Evans et al. [48] reported the identification of representatives of 80 different genera spanning 38 families, 17 separate orders, eight classes, and four major bacterial divisions: *Proteobacteria*, *Firmicutes*, *Actinobacteria*, and *Bacteroidete*. These authors argued that taxonomic diversities and patterns of abundance showed striking resemblance to those observed in freshwater and marine systems, and must reflect the presence of fully functional microbial ecosystems in domestic rainwater tanks. 

The fate of roof-water entering a storage tank needs to be set in the context of this microbiological complexity. After entry, untreated stored water undergoes a predictable sequence of physicochemical processes, which are outlined by Dixon et al. [49] as the settlement of suspended solids, aerobic microbial growth, anaerobic growth, and atmospheric re-aeration. The initial activity of aerobic microbes causes significant depletion of dissolved oxygen after 48 h, providing conditions favouring anaerobic growth. Consequent development of low pH through organic acid generation is likely to occur beyond this point, subject to availability of anaerobically digestible organic matter. Coates et al. [50] have shown that microbial re-oxidation of humic substances by anaerobic bacteria is environmentally ubiquitous. Microbes responsible were identified as belonging to alpha, beta, gamma, and delta subdivisions of the *Proteobacteria*, one of the four major bacterial divisions identified in Australian domestic roofwater tanks by Evans et al. [48].

Two further factors implicating dissolved organic acids as the most likely cause of the progressive increase in acidity were that no likely candidates could be found for inorganic acids that could have had the same impact, and that the development of low pH failed to occur in two tanks. This failure is consistent with the inhibition of a microbial process, for reasons suggested below.

##### Zn Concentrations, Minimum pH, and the Prevalence of *E. coli* in Each Tank

In Table 7, we list (in order of decreasing Zn concentration) the mean Zn concentrations, minimum pH, and the prevalence of *E. coli* in each tank.

The prevalence of unambiguous *E. coli* detections in this study was unusually low at 12.3%, which is at the low end of ranges reported in other studies. For example, Ahmed et al. [15] report a median level of prevalence of 57% (percent of samples reporting positive detections of *E. coli*) for nine studies from a range of countries. Spinks et al. [51] summarise five previous Australian tank water studies and report prevalence rates of 18%–38% *E. coli* detections. 

Fewtrell and Kay [52] list the main sources of external contamination of rainwater supplies as direct pollution from air (which may include microbes), bird and animal droppings, insects, and materials dissolved from the roofing material. There was no evidence to suggest an absence of conventional sources of *E. coli* to the water tanks studied in this investigation; for example, birdlife in the Wellington region of New Zealand is abundant. We note that unambiguous detection of *E. coli* only occurred in the two roof-fed tanks with the lowest concentrations of zinc (Table 6). In tanks with mean zinc ≥ 1.55 mg/L, *E. coli* was not detected. In parallel with this, the development of low pH (range 3.2–4.4) only occurred in the tanks with the lowest concentrations of zinc (<1.55 mg/L). This was not evident in either the two tanks with the highest mean zinc concentrations (Tanks 1 and 6), or the tapwater control (Tank 3) which would have contained residual free available chlorine from drinking-water disinfection. Over the six roof-fed tanks that were sampled, mean and median zinc (mg/L) are highly correlated with both minimum and 10th percentile pH (e.g., for mean Zn vs. minimum pH: *r* = 0.977, *p* < 0.001). 

##### Zinc Inhibition of Microbial Activity

As noted above, in tanks with the most zinc, low pH development did not occur, and microbial indicators were not detected (Figure 4, Table 5). These observations suggest that, at higher concentrations, zinc may cause significant inhibition of microbial activity, potentially including sterilisation. Available toxicity data for *E. coli* supports this idea. Li et al. [53] reported significant bacterial mortality in soft synthetic freshwater containing >0.2 mg/L free zinc, with typical figures being ~30% *E. coli* die-off at 0.5 mg/L zinc, rising to ~80% mortality at 2.5 mg/L zinc. Elevated calcium (Ca > 20 mg/L) or magnesium (Mg > 2 mg/L) reduced dissolved zinc toxicity, whereas sodium (Na) or potassium (K) had no impact; these results are relevant to this work because water in rain-fed tanks was soft, containing high Na but low Ca and Mg (Table 4). Comparison with the range of zinc concentrations in rain-fed tanks (Table 5) suggests that dissolved zinc would be predicted to cause substantial to total die-off of free *E. coli* in Tanks 1, 5 and 6 (mean zinc concentrations 4.45, 1.55, and 3.42 mg/L, respectively), and lesser but still significant bacterial mortality in Tanks 2 and 4 (0.85 and 0.58 mg/L, respectively). This fits with two observations in this work that (1) no *E. coli* detections occurred in Tanks 1, 5 or 6 and (2) overall detections were at the low end of the range reported across studies despite the presence of the usual sources. It is likely that, in tanks with high zinc, *E. coli* introduced in the normal way would have a limited survival time. Observations relating to pH can then be fully explained by assuming that the biocidal effects of high zinc extend beyond *E. coli* alone. Operationally, zinc has been trialled as an unconventional biocide to control bacterial problems in water distribution systems [54]. 

To conclude this section, we note that other studies have reported lower incidences of microbiological indicator species for tanks with galvanised steel catchments compared with other roofing materials, attributing this relationship to the combined effects of concentrated ultraviolet light and higher temperatures associated with galvanised steel roofing materials, providing a mechanism for sterilisation at source [14,28,29]. We have proposed here an alternative (or additional) hypothesis based on biocidal effects of high zinc concentrations, which occurs inside each tank rather than on the roof. This process is likely to cause partial to complete inhibition of a range of microbes across the tankwater microecosystem, including those involved in altering pH through a generation of organics acids. The observed relationships between zinc concentrations, pH, and *E. coli* prevalence support our hypothesis. 

Potential health implications of these findings are discussed in Section 4. 

#### 3.2.3. Lead

**Figure 9:**
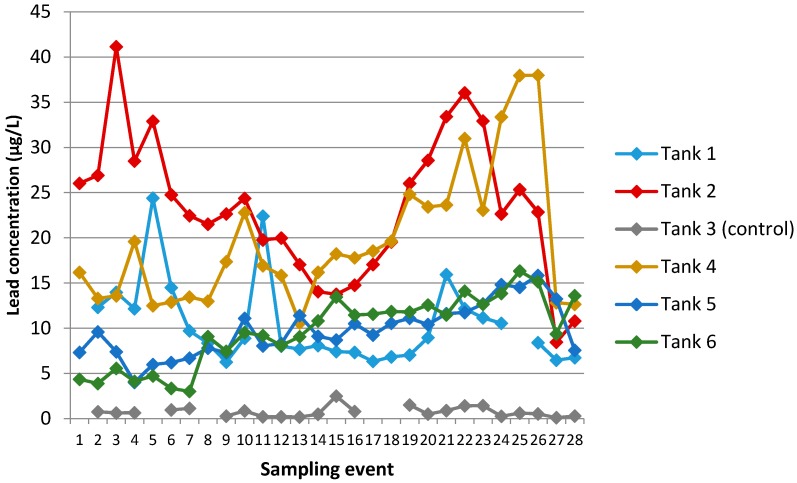
Changes in concentrations of lead over 28 sampling events.

Changes in concentrations of lead over time are shown in Figure 9.

Lead was enriched in all rain-fed tanks (range of 3–41 µg/L) compared with both a background rainwater sample (0.4 µg/L) and the tapwater control tank (mean of 0.7 µg/L, Table 4). Its appearance was not gradual, with all rain-fed tanks already showing lead enrichment from the first sampling event (Figure 9). Within each rain-fed tank, lead concentrations showed some (~threefold) variability with time but generally remained within a given order-of-magnitude range. The highest average and peak concentrations were found in Tanks 2 and 4. The roof catchments feeding these tanks were both comprised of old, painted galvanised steel. 

**Figure 10:**
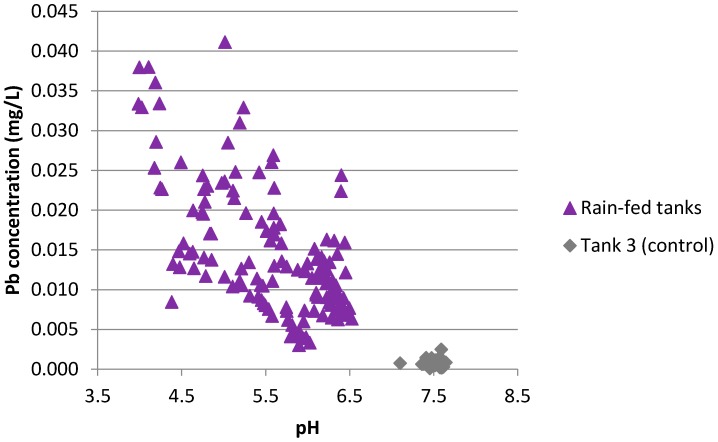
Relationship between pH and lead concentration for all samples.

Samples from all tanks (including the tapwater control) showed a highly significant linear relationship (*r* = −0.760, *p* < 0.0001) between pH and lead concentration (Figure 10). Omitting the control tank from the correlation yielded a weaker but still highly significant relationship. In individual tanks, linear relationships between pH and lead were found only for Tanks 4 and 5. 

In Tanks 4–6, lead concentrations increased (*p* < 0.0001) with time over the first year, but these trends did not persist into the second year (Figure 9). Of these, Tanks 5 and 6 were the two rain-fed tanks with the lowest initial lead readings, and Tank 6 showed the strongest linear correlation with time (*r* = 0.934). Over the first year, lead concentrations in Tank 6 increased by about 1 µg/L per month. We further note that, for the tanks in which lead concentrations increased over time, lead had strong positive linear correlations with zinc, sodium, and conductivity. In Section Trends in Zn Concentrations, we noted the strong linear correlations within all rain-fed tanks for sodium, zinc, and conductivity, and proposed that this may be due to the mobilisation of zinc as its aqueous chloride into first-flush rainfall runoff following a period of sea-salt deposition. It is possible that lead may also be mobilised in the same manner.

Observed trends in lead concentration (Figure 9) are likely to reflect the combination of the influence of an external source of lead together with a number of moderating factors such as a variable and possibly seasonal influence of corrosion chemistry [44], in-tank adsorption equilibria, evaporation, and water turnover (leading to an accumulation mechanism). The current data set does not allow us to quantify the relative magnitudes of each of these influences. 

Proposed mechanisms that may drive a general increase in concentrations in tanks over the first few months include both a log-normal input profile accompanied by restricted water turnover (as outlined for sodium and zinc in Section 3.1.2) and a secondary effect linked to sorption-equilibrium chemistry.

In relation to the second mechanism, when lead is present at µg/L (part per billion) concentrations in any non-acidified solution, adsorptive losses are expected to be both significant and noticeable. It is likely that a proportion of lead entering each tank is initially sequestered by adsorption to tank walls [41], bed sediments, and biofilms, gradually creating an increasing in-tank sink and reservoir of adsorbed lead. As the size of the adsorbed lead compartment increases, the lead concentration in tank water would in turn show a corresponding reflexive increase, proportionate to equilibrium partitioning of available lead in the connected reservoir. If this system is operative in some tanks, dissolved lead concentrations would be moderated by factors that perturb the adsorption equilibrium. Of these, acidity and dissolved organic complexing agents would be expected to have the greatest influence. 

An overall picture consistent with the lead data is that rainwater runoff takes on dissolved lead from each roof to a characteristic order-of-magnitude range (~10–40 µg/L). Higher lead concentrations are associated with older roof catchment systems and perhaps periods of higher sea-salt deposition and corrosion. After entry into each tank, some lead is adsorbed to solid materials (tank walls, biofilms, bottom sediments). The pool of adsorbed in-tank lead may then become a secondary reservoir for lead in the tank water by normal equilibrium partitioning. 

## 4. Discussion

### 4.1. Microbiological and Chemical Health Hazards

Rainwater and tapwater control tank results are shown in Table 8, in relation to both New Zealand [19] and international [55] drinking-water guidelines. While individual household supplies that serve less than 1500 person days (e.g., less than 25 persons for 60 days) do not have to comply with the DWSNZ, the standards still provide a convenient yardstick to determine the quality of the water in the emergency tanks. 

#### 4.1.1. Parameters of Health Significance

##### *E.* *coli*

In the rain-fed tanks, the overall rate of *E. coli* detections in this study was 17.7%, reducing to 12.3% if marginal detections (which are less strongly associated with health risks than clear detections) were excluded (Table 8). As an indicator of likely contamination of faecal origin, *E. coli* reflects the potential for water to be harbouring other, more pathogenic, organisms. Although the prevalence and magnitudes of *E. coli* detections in this study were at the low end of ranges reported internationally, the occasional presence of *E. coli* suggests that standard advice (provided on tank exteriors and reinforced by civil defence organisations) for consumers to disinfect water by boiling or adding chemical disinfectants prior to drinking is appropriate and justified. 

##### Lead

In the rain-fed tanks, 69% of the samples collected (*n* = 138) exceeded the maximum acceptable value (MAV) of 10 µg/L for Pb set by the DWSNZ (Table 8). Two of the five tanks were particularly high in lead, exceeding the MAV in 100% and 96% of samples. These two tanks were fed by the oldest roof catchment systems, with original painted galvanised steel roof cladding, in the study. These systems were also thought to have original lead head nails and flashing, but it was difficult to definitively identify these materials, much less quantify them. These older roof systems also contributed less zinc to tanks. They may have thus become more readily acidified (in line with our proposed hypothesis of zinc inhibition of microbial activity), which may have also contributed to the lead remaining in the tank water rather than adsorbed to the sediment within the tank or the tank walls. 

Lead cannot be removed from drinking water by boiling. Since the MAV for lead is assigned with long-term exposure in mind (Section 1.1.2), these results indicate unsuitability of such rain-fed tank water for routine use as a drinking-water supply. However, comparison to the MAV does not provide any index of potential risks of short-term consumption in an emergency situation. The World Health Organisation notes [55] that the exceedance of a health-based guideline value for short exposure periods may not result in an increased risk to health; therefore, the water may not necessarily be unfit for consumption. It is also important that water supplies not be unnecessarily restricted, as there is a direct relationship between access to adequate quantities of water and health risks due to requirements for hydration, food preparation, and basic hygiene. Even “basic access” (defined as ≤20 L/person/day, [56]), while sufficient to meet consumption needs, is still associated with a high level of health concern because the quantity of water available for measures such as handwashing, bathing, and basic food hygiene will be inadequate. 

Following a major earthquake on the Wellington Fault, the service outage period is likely to be at least 7 days (Section 1) and may be as long as 30 days or perhaps longer for a minority of households. We therefore suggest that there is a need for a more detailed health risk assessment relating to the risks of consuming water containing lead at the upper end of the observed range (~40 µg/L) over time periods of 7–30 days. At present, the World Health Organisation is in the process of developing health-based values for short-term exposures [55]. 

##### Other Elements and Compounds

For all 30 rain-fed tank water samples, concentrations of arsenic, cadmium, copper, and manganese remained below MAVs set by the DWSNZ (Table 8) and thus are not considered to be health risks either for short-term or long-term consumption.

Water samples from each tank were tested for 79 trace organic compounds by GC-MS. These compounds are indicative of a wide range of sources such as inputs from domestic woodburners, industrial and vehicle emissions, agricultural chemicals, and leaching from plastic tank materials. None of the target compounds were detected in water from any of the six tanks. This result implies that neither urban air pollution nor leaching from tank materials have any discernible effect on tank water quality. 

#### 4.1.2. Parameters of Aesthetic Significance

For all rain-fed tank water samples, pH values fell below the range recommended by DWSNZ (pH 7.0–8.5). This range is primarily intended to guide pH adjustment at water treatment plants, as pH is an important variable during the processes of coagulation and disinfection [17,19] and is also critically important for corrosion control. The low pH values observed in rain-fed tank water samples are not of direct health relevance, although they are indirectly relevant as lower pH values were associated with higher lead levels. 

A minor proportion (4.3%) of samples exceeded the DWSNZ GV of 2.5 NTU for turbidity. Above this level, water may be unacceptable to consumers because of visible cloudiness. Separate criteria for turbidity exist in the DWSNZ related to effects on disinfection. The minor exceedences in this study are thought to be primarily due to tank contents being disturbed on the few occasions where it was necessary to obtain samples by removing the lid of the tank due to the taps not working. 

For zinc, 52.9% of samples (*n* = 138) from rain-fed tanks exceeded the aesthetic guideline value of 1.5 mg/L. In Tanks 1 and 6 (both of which had unpainted galvanised roof cladding catchments), 100% of samples exceeded the GV. The World Health Organization [55] notes that water containing Zn in excess of ~3 mg/L may appear opalescent and develop a greasy film on boiling. If high Zn levels did cause the water to become unpalatable, there may be indirect health effects if the water supply to the household is restricted (Section 4.1.1). 

### 4.2. Management Recommendations for Tank Users

Firstly, our results on *E. coli* detections support the current management recommendation that all rain-fed tank water be boiled or otherwise disinfected prior to drinking. 

Secondly, we note that all samples drawn from a control tank filled at the outset of the study with Wellington reticulated tapwater complied with the DWSNZ for all parameters measured. This leads us to the recommendation that emergency rainwater tanks should be initially filled with tapwater, and allowed to refill with rainwater in the event of an emergency. The presence of residual free available chlorine in many treated municipal reticulated water supplies would also help discourage the growth of micro-organisms. 

Finally, we suggest cleaning tanks and refilling with tapwater on an annual basis, as in tanks with limited drawdown, contaminants such as lead and zinc can accumulate. Field observations from an accompanying report [43] also describe a build-up of biofilm and sediment over a year which may affect water palatability. 

### 4.3. Limitations of Study

Some aspects of the results described in this article may not apply to all rainwater tanks. The discussion applies specifically to (a) rainwater storage tanks, which experience low drawdown and recharge; (b) a coastal location with strong sea-salt aerosol influence; and (c) tanks which are relatively small (200 L, compared to more typical tank sizes of 5000 L used for supplying all household uses). Furthermore, the tanks in our study were of a basic design and did not have first flush diverters, which are a common feature of more sophisticated rainwater harvesting systems. 

Further limitations of this study are the relatively small number of roof catchment systems sampled, and that all roofs were clad with corrugated galvanised steel, both painted and unpainted. We note, however, that steel roofing is the most common roof cladding used in New Zealand, and was present on over half of all houses sampled in a comprehensive nationwide survey of building material type and condition [57]. Other cladding types commonly used in New Zealand are masonry tiles (on 28% of houses in same survey) and metal roof tiles (16%). 

As the study progressed, it became obvious that microbial processes were contributing to the evolution of the water chemistry in the rain-fed tanks, but the evidence remains circumstantial. Retrospectively, modifications could have been made to the study design to confirm and further characterize the changes attributable to microbial processes. Further studies could include direct measurements of dissolved oxygen (to confirm the onset of anaerobic conditions), 16S RNA sequencing to characterize the range of microbes present, and testing for the development of antibiotic resistance in tanks containing higher levels of dissolved zinc. 

## 5. Conclusions

To assess health hazards for householders in emergency situations, six 200-litre emergency water tanks were installed at properties across the Wellington region, with five tanks allowed to fill with roof-collected rainwater and one tank filled with municipal tapwater as a control. Sampling from these tanks was carried out fortnightly for one year, and samples were analysed for *E. coli,* pH, conductivity, a range of major and trace elements, and organic compounds, enabling an assessment of the evolution of water chemistry in water storage tanks over time. 

Considering all samples collected from the rain-fed tanks in this study in comparison to other studies on rainwater tank composition, in this study, pH values were very low (reaching levels of pH 4.0 in two tanks), conductivity was somewhat high (109 ± 55 µs/cm), and turbidity was low (0.8 ± 0.6 NTU). The prevalence of *E. coli* detections was at the low end of the range reported in other studies (17.7%, reducing to 12.3% if marginal detections were excluded). Of the major elements present at mg/L levels, sodium (13 ± 7 mg/L), zinc (2.1 ± 1.6 mg/L), and magnesium (1.8 ± 0.9 mg/L) were high in comparison to other studies, but calcium (1.2 ± 0.6 mg/L) was low. For the minor elements present at µg/L levels, lead (15 ± 8 µg/L) was high, but aluminium (6.3 ± 4.6 µg/L), arsenic (<1 µg/L), copper (6.6 ± 5.3 µg/L), cadmium (<0.05 µg/L), iron (<21 µg/L), and manganese (5.0 ± 3.3 µg/L) were low. Of the 79 organic compounds (from a wide range of sources including vehicle and industrial emissions and leaching from plastic tank materials) tested for, none were detected. This compositional profile reflects the coastal location of the study; the nature of the roof cladding, flashings, and fixings; and processes occurring within these emergency storage tanks. 

A novel feature of this study was that we obtained a year-long time series of data on chemical and microbiological water quality parameters to provide an insight into processes occurring within emergency rainwater storage tanks. For major chemical components, we identified a trend whereby concentrations initially increase via an “upward ratchet” mechanism; in the longer term, they will then oscillate around a plateau reflecting the mean of the concentration history. We also noted an association between zinc concentrations in tanks (determined by the nature of the roof cladding), the tendency of tanks to develop low pH levels, and the prevalence of *E. coli* in the tanks. While other studies have reported lower incidences of microbial indicator species for galvanised steel compared to other roofing materials, this has been attributed to the combined effects of concentrated ultraviolet light and higher temperatures on metal roofs providing a sterilising effect. We have proposed here an alternative hypothesis based on biocidal effects of high zinc concentrations. Finally, trends over time in lead concentrations were complex, and are thought to reflect external sources (particularly lead head nails and flashings), corrosion effects associated with sea-salt deposition, pH-related adsorption phenomena occurring with tanks, and the general accumulation mechanism described in Section 3.2.1. 

To identify chemical and microbiological health hazards associated with consumption of water from rain-fed tanks, values obtained in this study were compared with health-based and aesthetic guideline values set by the Drinking-Water Standards for New Zealand [19], which are very similar to the World Health Organisation’s Guidelines for Drinking-Water Quality [55]. Sixty-nine percent of rain-fed tank samples collected in this study exceeded the health-based guideline value for lead of 0.01 µg/L, indicating that this source is unsuitable for long-term consumption. In an emergency situation, however, assessing the health risk is more complex because of the lack of appropriate guideline values relevant to more short-term exposures. Use of the precautionary principle must be balanced against the recognition that, in an emergency, water supplies should not be unnecessarily restricted, as water shortages are associated with escalating health risks. The authors suggest that there is a need for a more detailed health risk assessment relating to the risks of consuming water at the upper end of the observed range of lead contamination (~40 µg/L, or four times above the long-term guideline) for exposure periods in the range of 7–30 days. A further health hazard was the detection of *E. coli* indicator bacteria in 17.7% of rain-fed tank samples. This is at the low end of the range reported by other studies, but nonetheless suggests that the standard advice for consumers to boil or otherwise sterilise roof-collected water supplies is well-justified. 

Concentrations of zinc were notably high in this study, and 53% of rain-fed tank samples exceeded the aesthetic guideline value of 1.5 mg/L, with all samples from two tanks exceeding this guideline. Any effects on water palatability could cause indirect health effects if the supply of potable water to the household is restricted. 

Our study supports the current recommendation that roof-collected rainwater be boiled or otherwise sterilised prior to drinking. New recommendations arising from this study are that emergency rainwater tanks should be initially filled with tapwater, and allowed to refill with rainwater in the event of an emergency, and that tanks should be cleaned and refilled with tapwater on an annual basis. 

## Figures and Tables

**Table 1:** Roof catchment characteristics at the study sites.

Site	Approximate Age of House	Roof Cladding Material and Condition	Lead Components Identified ^1^?	Home Heating Arrangements
1	1980 ^2^	Unpainted Galvanised Steel in Reasonable Condition	Yes	Woodburner as Primary Heating Source
2	1927	Old Painted Galvanised Steel in Poor Condition	No ^3^	No Solid Fuel Heating
4	1935	Old Painted Galvanised Steel in Poor Condition	Yes	No Solid Fuel Heating
5	1905	Areas of both New Unpainted Galvanised Steel and Old Unpainted Galvanised Steel	Yes	Woodburner as Primary Heating Source
6	1907	New Unpainted Galvanised Steel in very Good Condition	Yes	Woodburner as Secondary Heating Source

^1^ Lead components on roofs include lead head nails, ridge cap flashing, and chimney flashing. ^2^ At Site 1, the tank was connected to a garage roof rather than the main dwelling. ^3^ At this site, there was poor access to the roof and many areas could not be viewed.

**Table 2:** Summary of water quality parameters.

Parameter Measured	Sampling Frequency	Rationale for Inclusion
*E. coli*	Fortnightly	Inidicator Bacteria that is widely Used to Indicate the Risk of Faecal Contamination and Hence Pathogens.
pH	Fortnightly	Microbial Activity can Influence pH within Tank, which can then Influence Adsorption Behaviour of Metals.
Conductivity	Fortnightly	A measure of the Total Quantity of Dissolved Salts.
Major and Trace Elements	Once Every Two Months ^1^	Major Elements Reflect Influences such as Sea-Salt Deposition, which can Affect the Taste of the Water and Promote Corrosion. Minor Elements (Especially Copper, Lead, and Zinc) are primarily Contributed from the Components of the Roof Collection System and may be Important Contaminants in Drinking Water.
Semi-Volatile Organic Compounds (SVOCs)	Once a Year	SVOCs include Pesticide Residues, Polyaromatic Hydrocarbons from Incomplete Combusion, and Phthalates (used as Plasticisers). Many of these Compounds are of Toxicological Significance in Drinking Water and have Regulatory Limits Set.
BTEX ^2^	Once a Year	BTEX is the Term for a Group of Volatile Organic Compounds (Benzene, Toluene, Ethylbenzene, and Xylene) that are Found in Petroleum Products and Produced by Domestic Woodburners. They are of Toxicological Significance in Drinking Water and have Regulatory Limits set.

^1^ For the elements lead, zinc, and sodium, a complete data set was also obtained by retrospectively acidifying pH/conductivity samples with analysis by atomic absorption spectroscopy (AAS) (ContrAA 700, Analytik Jena, Jena, Germany) (see text). ^2^ Benzene, toluene, ethylbenzene, xylene.

**Table 3:** Summary of interlaboratory comparison results for sodium, lead, and zinc.

Statistic	Sodium	Zinc	Lead ^1^
Number of Common Samples	36	36	30
Mean RSD ^2^ between Labs (%) ^3^	3.0	4.6	15.5
Range of RSDs between Labs	0.2–9.5	0.0–17.1	0.5–58.2
Mean Apparent Recovery (AAS cf ICP-MS as %)	97.6	98.7	90.2

^1^ Tank 3 (which contained Wellington tap water) samples were omitted from the comparison as lead concentrations were close to detection limits. ^2^ Relative Standard Deviation. ^3^ Values cited are the mean of all relative standard deviations between all pairs of data for each element.

**Table 4:** Means and standard deviations (brackets) for measured variables in rainwater tanks. Sample numbers are given in footnotes.

Variable	Unit	Site 1	Site 2	Site 4	Site 5	Site 6	All 5 Rain-Fed Tanks	Site 3—Tapwater Control
pH	pH units	6.35 (0.13)	4.74 (0.43)	5.30 (0.58)	5.36 (0.50)	6.12 (0.16)	5.56 (0.71)	7.48 (0.14)
Conductivity	µS/cm	98.8 (18.8)	97.4 (31.1)	119 (42)	55.0 (13.1)	175 (64)	109 (55)	188 (6)
Turbidity	NTU	0.52 (0.18)	1.0 (0.84)	1.1 (0.71)	0.30 (0.17)	0.90 (0.51)	0.78 (0.63)	0.31 (0.10)
*E. coli*	MPN per 100 mL	<1	40.2 (168)	4.1 (12.5)	<1	<1	9.0 (76.4)	<1
Non-Purgeable Organic Carbon (NPOC) (mg/L)	mg/L	1.17 (0.23)	1.33 (0.39)	0.90 (0.25)	0.32 (0.26)	0.52 (0.42)	0.85 (0.49)	0.27 (0.22)
Calcium (Ca)	mg/L	1.63 (0.37)	1.04 (0.25)	1.12 (0.32)	0.55 (0.08)	1.66 (0.75)	1.20 (0.57)	19.4 (0.28)
Magnesium (Mg)	mg/L	1.51 (0.25)	1.82 (0.58)	2.05 (0.80)	0.78 (0.15)	2.68 (1.06)	1.77 (0.88)	2.78 (0.07)
Potassium (K)	mg/L	1.37 (0.30)	1.03 (0.35)	0.79 (0.36)	0.38 (0.10)	1.00 (0.44)	0.92 (0.45)	1.10 (0.07)
Sodium (Na)	mg/L	10.3 (2.0)	11.8 (4.0)	15.1 (5.2)	6.10 (1.56)	22.3 (8.5)	13.2 (7.3)	13.0 (0.4)
Zinc (Zn)	mg/L	4.45 (0.67)	0.852 (0.188)	0.584 (0.155)	1.55 (0.39)	3.42 (0.84)	2.14 (1.59)	0.063 (0.031)
Aluminum (Al)	µg/L	5.0 (0.7)	12.1 (5.5)	7.9 (2.0)	2.6 (2.0)	4.2 (4.0)	6.3 (4.6)	19.8 (1.4)
Arsenic (As)	µg/L	<1	<1	<1	4.8 (1.0)	<1	<1	<1
Cadmium (Cd)	µg/L	<0.05	<0.05	<0.05	0.09 (0.05)	<0.05	<0.05	<0.05
Copper (Cu)	µg/L	3.2 (2.3)	12.3 (8.2)	7.2 (4.2)	7.1 (2.8)	3.3 (1.2)	6.6 (5.3)	22.5 (1.5)
Iron (Fe)	µg/L	<21	<21	<21	<21	<21	<21	<21
Lead (Pb)	µg/L	10.5 (4.7)	23.4 (7.7)	19.6 (7.6)	9.7 (2.9)	9.7 (3.9)	14.6 (8.1)	0.7 (0.6)
Manganese (Mn)	µg/L	5.6 (0.6)	9.8 (4.7)	3.8 (1.3)	2.8 (0.7)	3.2 (1.1)	5.0 (3.3)	0.4 (0.4)

^1^ Sample numbers: in individual tanks: *n* = 26–28 for pH, conductivity, TDS, turbidity, Na, Pb, and Zn; *n* = 24–26 for *E. coli*; n = 6 for NPOC, Absorbance, Al, As, Ca, Cd, Cu, Fe, K, Mg, and Mn. Sample numbers across all five rain-fed tanks: *n* = 138 for pH, conductivity, TDS, turbidity, Na, Pb, and Zn; *n* = 128 for *E. coli*; *n* = 30 for NPOC, Absorbance, Al, As, Ca, Cd, Cu, Fe, K, Mg, and Mn. NTU = nephelometric turbidity units, MPN = most probable number.

**Table 5:** Extent to which increases in conductivity in each tank are described by linear relationships with time ^1^.

Tank	Slope of Line of Best Fit ^1^	R ^2^	Significance
1	1.29	0.570	*p* < 0.01
2	1.66	0.400	not significant
3	0.60	0.897	*p* < 0.0001
4	4.07	0.710	*p* < 0.001
5	1.31	0.981	*p* < 0.0001
6	7.63	0.944	*p* < 0.0001

^1^ Slope calculated for events 1–26 as (Δconductivity in µs/cm/Δsampling event number). ^2^ Pearson’s correlation coefficient.

**Table 6:** Linear correlations between conductivity, sodium, zinc, and pH (number of pairs 26–28).

Variable Pair (*N* = 26–28)	Direction of Relationship	*p*-Values					
		Tank 1	Tank 2	Tank 3	Tank 4	Tank 5	Tank 6
Conductivity with Sodium	positive	<0.0001	<0.0001	-	<0.0001	<0.0001	<0.0001
conductivity with Zinc	positive	<0.0001	<0.0001	<0.0001	<0.0001	<0.01	<0.0001
Sodium with Zinc	positive	<0.0001	<0.0001	-	<0.0001	<0.05 ^1^	<0.0001
pH with Zinc	negative	-	<0.01	-	<0.0001	<0.0001	-
Zinc with Time (First Year)	positive	<0.0001	<0.05	<0.0001	<0.0001	<0.0001	<0.0001
Sodium with Time (First Year)	positive	<0.0001	<0.05	<0.0001	<0.0001	<0.0001	<0.0001

^1^ This correlation improved to *p* < 0.0001 if the data from sampling event 28 was omitted.

**Table 7:** Apparent relationship between zinc concentrations and the viability of microorganisms as indicated by pH development and sampling events where *E. coli* was detected.

Tank Number	Roof Cladding Type	Mean Zinc Concentration (mg/L)	Minimum pH	Percent of Sampling Events *E. coli* Detected (%)
1	Unpainted Galvanised Steel	4.45	5.97	0
6	Unpainted Galvanised Steel	3.42	5.80	0
5	both Old Painted Galvanised Steel and New Unpainted Galvanised Steel	1.55	4.40	0
2	Old Painted Galvanised Steel	0.852	4.03	42
4	Old Painted Galvanised Steel	0.584	3.99	19
7 ^1^	Polycarbonate	0.162	3.19	(Not Measured)
3 (Tapwater Control)	Not Applicable (Not Plumbed)	0.063	7.05	0

^1^ Refer to Supplementary Table S5 for full details of results for Tank 7.

**Table 8:** Summary of study results in relation to drinking-water guidelines.

Parameter	DWSNZ ^1^	WHO ^2^	Proportion of Rainwater Samples Not Complying with DWSNZ ^3^	Proportion of Tapwater Control Samples Not Complying with DWSNZ ^3^
Parameters of Health Significance				
*E. coli*	<1 MPN/100 mL	<1 MPN/100 mL	17.7%	0%
Arsenic	0.01 mg/L	0.01 mg/L	0%	0%
Cadmium	0.004 mg/L	0.003 mg/L	0%	0%
Copper	2 mg/L	2 mg/L	0%	0%
Lead	0.01 mg/L	0.01 mg/L	69%	0%
Manganese	0.4 mg/L	-	0%	0%
Parameters of Aesthetic Significance				
pH	7.0–8.5	6.5–8.5	100%	0%
Turbidity	2.5 NTU	4 NTU	4.3%	0%
Hardness	200 mg/L	200 mg/L	0%	0%
Aluminium	0.1 mg/L	0.1–0.2 mg/L	0%	0%
Copper	1 mg/L	1 mg/L	0%	0%
Iron	0.2 mg/L	0.3 mg/L	0%	0%
Manganese	0.04 mg/L	0.1 mg/L	0%	0%
Sodium	200 mg/L	200 mg/L	0%	0%
Zinc	1.5 mg/L	3–5 mg/L	52.9%	0%

^1^ Drinking-Water Standards for New Zealand 2005 (revised 2008) [19]. ^2^ World Health Organisation Guidelines for Drinking-Water Quality Fourth Edition [55]. ^3^ Sample numbers given in Table 4.

## Supplementary Materials

The following are available online at http://www.mdpi.com/1660-4601/13/10/1012/s1, Table S1: Physicochemical and microbiological variables, Table S2: Results relating to trace elements present at ppm levels, Table S3: Results relating to trace elements present at ppm levels, Table S4: Identities and detection limits of organic compounds tested by gas chromatography-mass spectrometry (GC-MS), Table S5: Identities and detection limits of organic compounds tested by GC-MS, Table S6: Reconciliation of measured conductivities with major cation concentrations.
