# Supplementary material for: Health Hazards Associated with Consumption of Roof-Collected Rainwater in Urban Areas in Emergency Situations
Source: Int J Environ Res Public Health. 2016 Oct 15;13(10):1012. doi: 10.3390/ijerph13101012 (PMC5086751; doi:10.3390/ijerph13101012)
Supplement: Supplementary file 1 [file ijerph-13-01012-s001.docx]

Supplementary Materials: Health Hazards Associated with Consumption of Roof-Collected Rainwater in Urban Areas in Emergency Situations

Carol Stewart, Nick D. Kim, David M. Johnston and Mostafa Nayyerloo

1. Full Analytical Results

Full analytical results are provided in Tables S1–S5.

**Table S1.** Physicochemical and microbiological variables. *Interpretation:* Cond: conductivity; TDS: total dissolved solids (not independent, but calculated from conductivity assuming this is caused by sodium chloride); Turb: turbidity; *E. coli*: *Escherichia coli*; NPOC: Non-Purgeable Organic Carbon; Absorbance: light absorbed at the three wavelengths specified; Units: as specified; “**<**” sign indicates not detected at values above the detection limit specified. ***** Two samples considered to be sampling outliers, excluded from statistical analysis.

| **Sampling Date** | **Tank** | **pH** | **Cond** | **TDS** | **Turb** | ***E. coli*** | **NPOC** | **Absorbance (cm^−1^)** | | |
| --- | --- | --- | --- | --- | --- | --- | --- | --- | --- | --- |
|  |  |  | **µS/cm** | **mg/L** | **NTU** | **MPN/ 100mL** | **mg/L** | **340 nm** | **440 nm** | **780 nm** |
| 12 February 2014 * | 1 * | 7.12 * | 182.2 * | 91.2 * | 0.38 * | <1 * | - | - | - | - |
| 12 February 2014 | 2 | 5.57 | 64.5 | 32.4 | 0.98 | <1 | - | - | - | - |
| 12 February 2014 | 3 | 7.05 | 176.7 | 88.4 | 0.32 | <1 | - | - | - | - |
| 12 February 2014 | 4 | 6.32 | 74.6 | 37.4 | 1.00 | 3 | - | - | - | - |
| 12 February 2014 | 5 | 6.08 | 31.4 | 15.7 | 0.62 | <1 | - | - | - | - |
| 12 February 2014 | 6 | 5.85 | 79.1 | 39.6 | 1.29 | <1 | - | - | - | - |
| 26 February 2014 | 1 | 5.97 | 85.3 | 42.6 | 0.36 | <1 | - | - | - | - |
| 26 February 2014 | 2 | 5.59 | 74.3 | 37.2 | 0.67 | <1 | - | - | - | - |
| 26 February 2014 | 3 | 7.10 | 185.2 | 92.7 | 0.28 | <1 | - | - | - | - |
| 26 February 2014 | 4 | 6.00 | 90.0 | 45.0 | 0.70 | <1 | - | - | - |  |
| 26 February 2014 | 5 | 6.10 | 35.2 | 17.6 | 0.67 | <1 | - | - | - | - |
| 26 February 2014 | 6 | 5.95 | 92.4 | 46.2 | 0.89 | <1 | - | - | - | - |
| 12 March 2014 | 1 | 6.14 | 102.9 | 51.5 | 0.55 | <1 | 1.5 | 0.013 | 0.003 | <0.002 |
| 12 March 2014 | 2 | 5.02 | 114.0 | 57.0 | 0.96 | <1 | 1.7 | 0.022 | 0.004 | <0.002 |
| 12 March 2014 | 3 | 7.36 | 185.9 | 93.0 | 0.55 | <1 | <0.3 | <0.002 | <0.002 | <0.002 |
| 12 March 2014 | 4 | 5.69 | 104.2 | 52.1 | 0.78 | <1 | 0.8 | 0.01 | <0.002 | <0.002 |
| 12 March 2014 | 5 | 5.96 | 37.2 | 18.6 | 0.36 | <1 | <0.3 | 0.002 | <0.002 | <0.002 |
| 12 March 2014 | 6 | 5.82 | 113.4 | 56.7 | 0.95 | <1 | <0.3 | <0.002 | <0.002 | <0.002 |
| 26 March 2014 | 1 | 6.14 | 104.5 | 52.3 | 0.83 | <1 | - | - | - | - |
| 26 March 2014 | 2 | 5.05 | 105.2 | 52.6 | 1.30 | 4 | - | - | - | - |
| 26 March 2014 | 3 | 7.38 | 179.0 | 89.6 | 0.58 | <1 | - | - | - | - |
| 26 March 2014 | 4 | 5.60 | 106.3 | 53.2 | 1.64 | 1 | - | - | - | - |
| 26 March 2014 | 5 | 5.98 | 34.9 | 17.4 | 0.50 | <1 | - | - | - | - |
| 26 March 2014 | 6 | 5.80 | 109.5 | 54.8 | 1.68 | <1 | - | - | - | - |
| 9 April 2014 | 1 | 6.40 | 114.8 | 57.4 | 0.78 | <1 | - | - | - | - |
| 9 April 2014 | 2 | 5.24 | 103.9 | 52.0 | 0.77 | 56 | - | - | - | - |
| 9 April 2014 | 3 | 7.55 | 183.0 | 91.6 | 0.31 | <1 | - | - | - | - |
| 9 April 2014 | 4 | 5.88 | 87.9 | 44.0 | 0.69 | 58 | - | - | - | - |
| 9 April 2014 | 5 | 5.95 | 36.7 | 18.3 | 0.42 | 2 | - | - | - | - |
| 9 April 2014 | 6 | 5.90 | 104.5 | 52.3 | 0.90 | <1 | - | - | - | - |
| 23 April 2014 | 1 | 6.35 | 82.0 | 41.0 | 0.59 | <1 | - | - | - | - |
| 23 April 2014 | 2 | 5.42 | 75.7 | 37.9 | 0.65 | 6 | - | - | - | - |
| 23 April 2014 | 3 | 7.47 | 183.7 | 91.9 | 0.35 | <1 | - | - | - | - |
| 23 April 2014 | 4 | 5.75 | 51.2 | 25.6 | 0.53 | 1 | - | - | - | - |
| 23 April 2014 | 5 | 5.77 | 45.6 | 22.8 | 0.59 | <1 | - | - | - | - |
| 23 April 2014 | 6 | 6.03 | 88.3 | 44.1 | 0.84 | <1 | - | - | - | - |
| 7 May 2014 | 1 | 6.32 | 75.8 | 37.9 | 0.72 | <1 | 1.1 | 0.011 | 0.003 | <0.002 |
| 7 May 2014 | 2 | 5.11 | 76.3 | 38.2 | 0.72 | 860 | 1.6 | 0.019 | 0.004 | <0.002 |
| 7 May 2014 | 3 | 7.52 | 186.0 | 93.1 | 0.35 | <1 | 0.4 | <0.002 | <0.002 | <0.002 |
| 7 May 2014 | 4 | 5.30 | 61.5 | 30.7 | 0.55 | <1 | 0.8 | 0.007 | <0.002 | <0.002 |
| 7 May 2014 | 5 | 5.58 | 48.0 | 24.0 | 0.50 | <1 | 0.4 | 0.003 | <0.002 | <0.002 |
| 7 May 2014 | 6 | 5.90 | 97.8 | 48.9 | 1.02 | <1 | <0.3 | <0.002 | <0.002 | <0.002 |
| 21 May 2014 | 1 | 6.39 | 71.9 | 36.0 | 0.69 | <1 | - | - | - | - |
| 21 May 2014 | 2 | 5.13 | 66.6 | 33.3 | 0.50 | 26 | - | - | - | - |
| 21 May 2014 | 3 | 7.62 | 182.8 | 91.4 | 0.29 | <1 | - | - | - | - |
| 21 May 2014 | 4 | 5.60 | 51.6 | 25.8 | 0.43 | <1 | - | - | - | - |
| 21 May 2014 | 5 | 5.75 | 48.2 | 24.1 | 0.37 | <1 | - | - | - | - |
| 21 May 2014 | 6 | 6.12 | 97.5 | 48.7 | 0.84 | <1 | - | - | - | - |
| 4 June 2014 | 1 | 6.36 | 93.9 | 47.0 | 0.35 | <1 | - | - | - | - |
| 4 June 2014 | 2 | 4.77 | 97.4 | 48.8 | 0.43 | 9 | - | - | - | - |
| 4 June 2014 | 3 | 7.62 | 184.8 | 92.4 | 0.22 | <1 | - | - | - | - |
| 4 June 2014 | 4 | 5.51 | 149.6 | 74.8 | 0.55 | <1 | - | - | - | - |
| 4 June 2014 | 5 | 5.75 | 51.7 | 25.8 | 0.16 | <1 | - | - | - | - |
| 4 June 2014 | 6 | 6.35 | 157.7 | 78.9 | 0.73 | <1 | - | - | - | - |
| 18 June 2014 | 1 | 6.37 | 89.5 | 44.7 | 0.42 | <1 | - | - | - | - |
| 18 June 2014 | 2 | 4.75 | 84.6 | 42.3 | 0.46 | 21 | - | - | - | - |
| 18 June 2014 | 3 | 7.645 | 184.7 | 92.4 | 0.34 | <1 | - | - | - | - |
| 18 June 2014 | 4 | 5.6 | 115.6 | 57.8 | 0.51 | 1 | - | - | - | - |
| 18 June 2014 | 5 | 5.58 | 51.8 | 25.9 | 0.29 | <1 | - | - | - | - |
| 18 June 2014 | 6 | 6.36 | 138.4 | 69.2 | 0.74 | <1 | - | - | - | - |
| 2 July 2014 | 1 | 6.40 | 82.0 | 41.0 | 0.50 | <1 | 1.2 | 0.009 | 0.002 | <0.002 |
| 2 July 2014 | 2 | 4.75 | 66.0 | 33.0 | 0.44 | 3 | 1.4 | 0.01 | <0.002 | <0.002 |
| 2 July 2014 | 3 | 7.58 | 181.5 | 90.8 | 0.19 | <1 | 0.5 | <0.002 | <0.002 | <0.002 |
| 2 July 2014 | 4 | 5.60 | 97.7 | 48.9 | 0.56 | <1 | 0.9 | 0.006 | <0.002 | <0.002 |
| 2 July 2014 | 5 | 5.50 | 51.5 | 25.8 | 0.19 | <1 | 0.5 | <0.002 | <0.002 | <0.002 |
| 2 July 2014 | 6 | 6.26 | 134.0 | 67.0 | 0.65 | <1 | 0.7 | <0.002 | <0.002 | <0.002 |
| 16 July 2014 | 1 | 6.41 | 82.4 | 41.2 | 0.42 | <1 | - | - | - | - |
| 16 July 2014 | 2 | 4.64 | 70.7 | 35.3 | 0.71 | 1 | - | - | - | - |
| 16 July 2014 | 3 | 7.59 | 187.9 | 94.0 | 0.23 | <1 | - | - | - | - |
| 16 July 2014 | 4 | 5.69 | 94.6 | 47.3 | 0.66 | <1 | - | - | - | - |
| 16 July 2014 | 5 | 5.48 | 50.7 | 25.4 | 0.15 | <1 | - | - | - | - |
| 16 July 2014 | 6 | 6.28 | 138.0 | 69.0 | 0.54 | <1 | - | - | - | - |
| 30 July 2014 | 1 | 6.50 | 84.0 | 42.0 | 0.36 | <1 | - | - | - | - |
| 30 July 2014 | 2 | 4.85 | 65.5 | 32.8 | 0.72 | <1 | - | - | - | - |
| 30 July 2014 | 3 | 7.60 | 186.1 | 93.1 | 0.26 | <1 | - | - | - | - |
| 30 July 2014 | 4 | 5.44 | 66.1 | 33.1 | 1.39 | <1 | - | - | - | - |
| 30 July 2014 | 5 | 5.40 | 57.0 | 28.5 | 0.16 | <1 | - | - | - | - |
| 30 July 2014 | 6 | 6.23 | 137.8 | 68.9 | 2.12 | <1 | - | - | - | - |
| 13 August 2014 | 1 | 6.39 | 83.9 | 41.9 | 0.41 | <1 | - | - | - | - |
| 13 August 2014 | 2 | 4.77 | 62.0 | 31.0 | 3.09 | <1 | - | - | - | - |
| 13 August 2014 | 3 | 7.52 | 184.8 | 92.4 | 0.25 | <1 | - | - | - | - |
| 13 August 2014 | 4 | 5.56 | 84.2 | 42.1 | 0.85 | <1 | - | - | - | - |
| 13 August 2014 | 5 | 5.41 | 57.0 | 28.5 | 0.18 | <1 | - | - | - | - |
| 13 August 2014 | 6 | 6.22 | 148.2 | 74.1 | 1.21 | <1 | - | - | - | - |
| 27 August 2014 | 1 | 6.44 | 87.6 | 43.8 | 0.49 | <1 | 0.8 | 0.009 | 0.002 | <0.002 |
| 27 August 2014 | 2 | 4.855 | 97.3 | 48.7 | 2.84 | <1 | 0.6 | 0.006 | <0.002 | <0.002 |
| 27 August 2014 | 3 | 7.59 | 186.6 | 93.3 | 0.32 | <1 | 0.3 | <0.002 | <0.002 | <0.002 |
| 27 August 2014 | 4 | 5.67 | 113.0 | 56.5 | 2.82 | <1 | 0.7 | 0.007 | <0.002 | <0.002 |
| 27 August 2014 | 5 | 5.45 | 57.4 | 28.7 | 0.20 | <1 | 0.4 | <0.002 | <0.002 | <0.002 |
| 27 August 2014 | 6 | 6.26 | 183.0 | 91.6 | 1.41 | <1 | 1.0 | <0.002 | <0.002 | <0.002 |
| 10 September 2014 | 1 | 6.42 | 93.4 | 46.7 | 0.99 | <1 | - | - | - | - |
| 10 September 2014 | 2 | 4.63 | 98.4 | 49.2 | 1.41 | <1 | - | - | - | - |
| 10 September 2014 | 3 | 7.51 | 188.2 | 94.2 | 0.44 | <1 | - | - | - | - |
| 10 September 2014 | 4 | 5.60 | 100.0 | 50.0 | 1.18 | <1 | - | - | - | - |
| 10 September 2014 | 5 | 5.47 | 58.0 | 29.0 | 0.25 | <1 | - | - | - | - |
| 10 September 2014 | 6 | 6.16 | 178.0 | 89.0 | 2.52 | <1 | - | - | - | - |
| 24 September 2014 | 1 | 6.53 | 97.6 | 48.8 | 0.31 | <1 | - | - | - | - |
| 24 September 2014 | 2 | 4.84 | 132.8 | 66.4 | 1.69 | 2 | - | - | - | - |
| 24 September 2014 | 3 | 7.60 | 188.0 | 94.0 | 0.28 | <1 | - | - | - | - |
| 24 September 2014 | 4 | 5.45 | 173.2 | 86.6 | 1.30 | 30 | - | - | - | - |
| 24 September 2014 | 5 | 5.31 | 58.2 | 29.1 | 0.16 | <1 | - | - | - | - |
| 24 September 2014 | 6 | 6.23 | 220.0 | 110.0 | 0.78 | <1 | - | - | - | - |
| 8 October 2014 | 1 | 6.46 | 113.8 | 56.9 | 0.47 | <1 | - | - | - | - |
| 8 October 2014 | 2 | 4.76 | 140.4 | 70.2 | 3.64 | 4 | - | - | - | - |
| 8 October 2014 | 3 | 7.57 | 188.6 | 94.3 | 0.50 | <1 | - | - | - | - |
| 8 October 2014 | 4 | 5.27 | 183.0 | 91.5 | 0.97 | <1 | - | - | - | - |
| 8 October 2014 | 5 | 5.22 | 58.6 | 29.3 | 0.20 | <1 | - | - | - | - |
| 8 October 2014 | 6 | 6.20 | 225.0 | 112.0 | 0.98 | <1 | - | - | - | - |
| 22 October 2014 | 1 | 6.47 | 117.4 | 58.7 | 0.84 | <1 | 1.1 | 0.011 | 0.003 | <0.002 |
| 22 October 2014 | 2 | 4.49 | 146.9 | 73.5 | 1.36 | <1 | 1.3 | 0.019 | 0.004 | <0.002 |
| 22 October 2014 | 3 | 7.57 | 189.6 | 94.8 | 0.29 | <1 | <0.3 | <0.002 | <0.002 | <0.002 |
| 22 October 2014 | 4 | 5.14 | 187.0 | 93.5 | 1.03 | <1 | 0.8 | 0.010 | <0.002 | <0.002 |
| 22 October 2014 | 5 | 5.19 | 62.2 | 31.1 | 0.34 | <1 | <0.3 | <0.002 | <0.002 | <0.002 |
| 22 October 2014 | 6 | 6.21 | 235.0 | 118 | 0.76 | <1 | 0.6 | 0.002 | <0.002 | <0.002 |
| 5 November 2014 | 1 | 6.43 | 120.4 | 60.2 | 0.38 | <1 | - | - | - | - |
| 5 November 2014 | 2 | 4.20 | 153.2 | 76.6 | 0.48 | <1 | - | - | - | - |
| 5 November 2014 | 3 | 7.51 | 191.0 | 95.6 | 0.33 | <1 | - | - | - | - |
| 5 November 2014 | 4 | 4.98 | 176.6 | 88.4 | 1.26 | 4 | - | - | - | - |
| 5 November 2014 | 5 | 5.11 | 60.6 | 30.3 | 0.20 | <1 | - | - | - | - |
| 5 November 2014 | 6 | 6.23 | 242.0 | 121.0 | 0.52 | <1 | - | - | - | - |
| 19 November 2014 | 1 | 6.44 | 120.2 | 60.1 | 0.56 | <1 | - | - | - | - |
| 19 November 2014 | 2 | 4.24 | 158.0 | 79.0 | 0.70 | <1 | - | - | - | - |
| 19 November 2014 | 3 | 7.42 | 192.0 | 96.0 | 0.33 | <1 | - | - | - | - |
| 19 November 2014 | 4 | 5.01 | 163.7 | 81.9 | 1.36 | <1 | - | - | - | - |
| 19 November 2014 | 5 | 5.01 | 61.6 | 30.8 | 0.18 | <1 | - | - | - | - |
| 19 November 2014 | 6 | 6.05 | 245.0 | 123.0 | 0.73 | <1 | - | - | - | - |
| 3 December 2014 | 1 | 6.45 | 117.0 | 58.5 | 0.45 | <1 | - | - |  | - |
| 3 December 2014 | 2 | 4.19 | 139.9 | 70.0 | 0.68 | <1 | - | - | - | - |
| 3 December 2014 | 3 | 7.48 | 191.7 | 95.9 | 0.33 | <1 | - | - | - | - |
| 3 December 2014 | 4 | 5.19 | 181.6 | 90.9 | 3.72 | 8 | - | - | - | - |
| 3 December 2014 | 5 | 4.79 | 62.7 | 31.3 | 0.16 | <1 | - | - | - | - |
| 3 December 2014 | 6 | 6.17 | 258.0 | 129.0 | 0.52 | <1 | - | - | - | - |
| 17 December 2014 | 1 | 6.30 | 117.2 | 58.6 | 0.28 | <1 | 1.30 | 0.013 | 0.002 | <0.002 |
| 17 December 2014 | 2 | 4.03 | 136.9 | 68.5 | 0.47 | <1 | 1.40 | 0.016 | <0.002 | <0.002 |
| 17 December 2014 | 3 | 7.41 | 194.5 | 97.3 | 0.30 | <1 | 0.40 | <0.002 | <0.002 | <0.002 |
| 17 December 2014 | 4 | 4.81 | 159.6 | 79.8 | 1.71 | <1 | 1.40 | 0.009 | <0.002 | <0.002 |
| 17 December 2014 | 5 | 4.65 | 62.8 | 31.4 | 0.18 | <1 | 0.60 | <0.002 | <0.002 | <0.002 |
| 17 December 2014 | 6 | 6.15 | 256.0 | 128.0 | 0.54 | - | - | - | - | <0.002 |
| 14 January 2015 | 1 | 6.34 | 103.2 | 51.6 | 0.43 | <1 | - | - | - | - |
| 14 January 2015 | 2 | 4.27 | 75.8 | 37.9 | 0.59 | 50 | - | - | - | - |
| 14 January 2015 | 3 | 7.46 | 194.4 | 97.3 | 0.32 | <1 | - | - | - | - |
| 14 January 2015 | 4 | 3.99 | 138.9 | 69.5 | 0.78 | <1 | - | - | - | - |
| 14 January 2015 | 5 | 4.47 | 64.5 | 32.2 | 0.22 | <1 | - | - | - | - |
| 14 January 2015 | 6 | 6.11 | 230.0 | 115.0 | 0.45 | <1 | - | - | - | - |
| 28 January 2015 * | 1 * | 7.24 * | 186.7 * | 93.4 * | 0.62 * | <1 * | - | - | - | - |
| 28 January 2015 | 2 | 4.18 | 77.3 | 38.7 | 0.45 | 4 | - | - | - | - |
| 28 January 2015 | 3 | 7.47 | 195.9 | 98.0 | 0.32 | <1 | - | - | - | - |
| 28 January 2015 | 4 | 4.00 | 142.0 | 71.0 | 0.89 | <1 | - | - | - | - |
| 28 January 2015 | 5 | 4.60 | 64.8 | 32.4 | 0.20 | <1 | - | - | - | - |
| 28 January 2015 | 6 | 6.23 | 230.0 | 115.0 | 0.46 | <1 | - | - | - | - |
| 11 February 2015 | 1 | 6.26 | 117.2 | 58.6 | 0.44 | 2 | - | - | - | - |
| 11 February 2015 | 2 | 4.25 | 76.2 | 38.1 | 0.45 | <1 | - | - | - | - |
| 11 February 2015 | 3 | 7.43 | 198.0 | 99.0 | 0.22 | <1 | - | - | - | - |
| 11 February 2015 | 4 | 4.11 | 151.0 | 75.5 | 0.99 | <1 | - | - | - | - |
| 11 February 2015 | 5 | 4.52 | 66.6 | 33.3 | 0.17 | <1 | - | - | - | - |
| 11 February 2015 | 6 | 6.08 | 240.0 | 120.0 | 0.49 | <1 | - | - | - | - |
| 2 April 2015 | 1 | 6.29 | 143.9 | 72.0 | 0.51 | - | - | - | - | - |
| 2 April 2015 | 2 | 4.38 | 65.0 | 32.5 | 0.38 | - | - | - | - | - |
| 2 April 2015 | 3 | 7.46 | 201.0 | 101.0 | 0.21 | - | - | - | - | - |
| 2 April 2015 | 4 | 4.48 | 128.4 | 64.2 | 0.76 | - | - | - | - | - |
| 2 April 2015 | 5 | 4.40 | 68.2 | 34.1 | 0.21 | - | - | - | - | - |
| 2 April 2015 | 6 | 6.10 | 265.0 | 133.0 | 0.40 | - | - | - | - | - |
| 1 July 2015 | 1 | 6.18 | 67.5 | 33.7 | 0.34 | - | - | - | - | - |
| 1 July 2015 | 2 | 4.78 | 101.7 | 50.8 | 1.03 | - | - | - | - | - |
| 1 July 2015 | 3 | 7.44 | 198.2 | 99.1 | 0.15 | - | - | - | - | - |
| 1 July 2015 | 4 | 5.21 | 97.6 | 48.8 | 1.07 | - | - | - | - | - |
| 1 July 2015 | 5 | 5.54 | 95.5 | 47.7 | 0.76 | - | - | - | - | - |
| 1 July 2015 | 6 | 6.20 | 243.0 | 122.0 | 0.37 | - | - | - | - | - |

**Table S2.** Results relating to trace elements present at ppm levels. *Interpretation:* Units: all concentrations in mg/L (g/m^3^) (parts per million); Ca: total calcium measured by ICP-MS; Mg: total magnesium measured by ICP-MS; K: total potassium measured by ICP-MS; Na: total sodium measured by ICP-MS; Na AAS: acid recoverable sodium measured by Atomic Absorption Spectroscopy; Zn: total zinc measured by ICP-MS; Zn AAS: acid recoverable zinc measured by Atomic Absorption Spectroscopy; “<” sign indicates not detected at concentrations above the figure specified. * Two samples considered to be sampling outliers.

| **Sampling Date** | **Tank** | **Ca** | **Mg** | **K** | **Na** | **Na AAS** | **Zn** | **Zn AAS** |
| --- | --- | --- | --- | --- | --- | --- | --- | --- |
| 12 February 2014 | 1 | - | - | - | - | * | - | * |
| 12 February 2014 | 2 | - | - | - | - | 7.82 | - | 0.694 |
| 12 February 2014 | 3 | - | - | - | - | 12.52 | - | 0.045 |
| 12 February 2014 | 4 | - | - | - | - | 10.28 | - | 0.324 |
| 12 February 2014 | 5 | - | - | - | - | 3.39 | - | 0.91 |
| 12 February 2014 | 6 | - | - | - | - | 10.49 | - | 2.01 |
| 26 February 2014 | 1 | - | - | - | - | 8.23 | - | 4.01 |
| 26 February 2014 | 2 | - | - |  | - | 8.24 | - | 0.771 |
| 26 February 2014 | 3 | - | - | - | - | 12.80 | - | 0.018 |
| 26 February 2014 | 4 | - | - | - | - | 11.66 | - | 0.446 |
| 26 February 2014 | 5 | - | - | - | - | 3.46 | - | 0.88 |
| 26 February 2014 | 6 | - | - | - | - | 10.92 | - | 2.45 |
| 12 March 2014 | 1 | 1.38 | 1.57 | 1.57 | 10.6 | 10.36 | 4.6 | 4.16 |
| 12 March 2014 | 2 | 1.29 | 1.87 | 1.29 | 12.8 | 14.18 | 0.98 | 0.978 |
| 12 March 2014 | 3 | 19.2 | 2.7 | 1.08 | 12.4 | 12.88 | 0.028 | 0.028 |
| 12 March 2014 | 4 | 1.06 | 1.65 | 0.64 | 12.7 | 13.74 | 0.52 | 0.481 |
| 12 March 2014 | 5 | 0.43 | 0.5 | 0.21 | 3.7 | 3.64 | 0.98 | 0.91 |
| 12 March 2014 | 6 | 0.76 | 1.73 | 0.57 | 13.5 | 13.66 | 2.6 | 2.68 |
| 26 March 2014 | 1 | - | - | - | - | 10.70 | - | 3.95 |
| 26 March 2014 | 2 | - | - | - | - | 13.57 | - | 0.872 |
| 26 March 2014 | 3 | - | - | - | - | 12.97 | - | 0.018 |
| 26 March 2014 | 4 | - | - | - | - | 14.30 | - | 0.535 |
| 26 March 2014 | 5 | - | - | - | - | 3.81 | - | 0.97 |
| 26 March 2014 | 6 | - |  |  |  | 13.55 |  | 2.77 |
| 9 April 2014 | 1 | - | - | - | - | 12.63 | - | 4.26 |
| 9 April 2014 | 2 | - | - | - | - | 13.83 | - | 0.910 |
| 9 April 2014 | 3 | - | - | - | - | 12.70 | - | 0.073 |
| 9 April 2014 | 4 | - | - | - | - | 11.69 | - | 0.452 |
| 9 April 2014 | 5 | - | - | - | - | 4.05 | - | 1.01 |
| 9 April 2014 | 6 | - | - | - | - | 13.05 | - | 2.58 |
| 23 April 2014 | 1 | - | - | - | - | 8.30 | - | 3.57 |
| 23 April 2014 | 2 | - | - | - | - | 9.15 | - | 0.651 |
| 23 April 2014 | 3 | - | - | - | - | 12.97 | - | 0.026 |
| 23 April 2014 | 4 | - | - | - | - | 6.67 | - | 0.322 |
| 23 April 2014 | 5 | - | - | - | - | 5.21 | - | 1.27 |
| 23 April 2014 | 6 | - | - | - | - | 11.65 | - | 2.31 |
| 7 May 2014 | 1 | 1.31 | 1.22 | 1.05 | 8.4 | 8.12 | 3.9 | 3.40 |
| 7 May 2014 | 2 | 0.93 | 1.34 | 0.86 | 9.5 | 9.37 | 0.73 | 0.678 |
| 7 May 2014 | 3 | 19.4 | 2.8 | 1.04 | 13.2 | 12.95 | 0.037 | 0.032 |
| 7 May 2014 | 4 | 0.74 | 1.05 | 0.36 | 8.3 | 8.15 | 0.42 | 0.340 |
| 7 May 2014 | 5 | 0.51 | 0.71 | 0.35 | 6.2 | 5.42 | 1.43 | 1.37 |
| 7 May 2014 | 6 | 0.76 | 1.56 | 0.54 | 13.0 | 12.05 | 2.6 | 2.50 |
| 21 May 2014 | 1 | - | - | - | - | 7.32 | - | 3.36 |
| 21 May 2014 | 2 | - | - | - | - | 8.01 | - | 0.607 |
| 21 May 2014 | 3 | - | - | - | - | 13.16 | - | 0.044 |
| 21 May 2014 | 4 | - | - | - | - | 6.61 | - | 0.328 |
| 21 May 2014 | 5 | - | - | - | - | 5.56 | - | 1.40 |
| 21 May 2014 | 6 | - | - | - | - | 11.81 | - | 2.57 |
| 4 June 2014 | 1 | - | - | - | - | 10.19 | - | 3.80 |
| 4 June 2014 | 2 | - | - | - | - | 12.49 |  | 0.818 |
| 4 June 2014 | 3 | - | - | - | - | 13.51 | - | 0.025 |
| 4 June 2014 | 4 | - | - | - | - | 21.51 | - | 0.752 |
| 4 June 2014 | 5 | - | - | - | - | 5.89 | - | 1.51 |
| 4 June 2014 | 6 | - | - | - | - | 20.12 | - | 2.83 |
| 18 June 2014 | 1 | - | - | - | - | 9.63 | - | 4.07 |
| 18 June 2014 | 2 | - | - | - | - | 10.35 | - | 0.810 |
| 18 June 2014 | 3 | - | - |  | - | 13.21 | - | 0.037 |
| 18 June 2014 | 4 | - | - | - | - | 16.16 | - | 0.653 |
| 18 June 2014 | 5 | - | - | - | - | 5.89 | - | 1.59 |
| 18 June 2014 | 6 | - | - | - | - | 17.07 | - | 2.90 |
| 2 July 2014 | 1 | 1.438 | 1.31 | 1.172 | 9.24 | 8.74 | 4.37 | 4.16 |
| 2 July 2014 | 2 | 0.773 | 1.121 | 0.644 | 8.24 | 8.14 | 0.769 | 0.745 |
| 2 July 2014 | 3 | 19.08 | 2.77 | 1.06 | 13.63 | 13.34 | 0.046 | 0.036 |
| 2 July 2014 | 4 | 0.977 | 1.71 | 0.608 | 14.38 | 13.76 | 0.701 | 0.656 |
| 2 July 2014 | 5 | 0.557 | 0.808 | 0.399 | 6.55 | 5.94 | 1.6 | 1.64 |
| 2 July 2014 | 6 | 1.702 | 2.07 | 0.759 | 18.5 | 16.49 | 3.05 | 3.00 |
| 16 July 2014 | 1 | - | - | - |  | 8.60 | - | 4.01 |
| 16 July 2014 | 2 | - | - | - |  | 8.26 | - | 0.733 |
| 16 July 2014 | 3 | - | - | - |  | 13.48 | - | 0.040 |
| 16 July 2014 | 4 | - | - | - |  | 12.81 | - | 0.641 |
| 16 July 2014 | 5 | - | - | - |  | 6.03 | - | 1.51 |
| 16 July 2014 | 6 | - | - | - | - | 17.38 | - | 3.19 |
| 30 July 2014 | 1 | - | - | - | - | 8.76 | - | 4.14 |
| 30 July 2014 | 2 | - | - | - | - | 8.34 | - | 0.655 |
| 30 July 2014 | 3 | - | - | - | - | 13.46 | - | 0.049 |
| 30 July 2014 | 4 | - | - | - | - | 9.05 | - | 0.350 |
| 30 July 2014 | 5 | - | - | - | - | 6.26 | - | 1.64 |
| 30 July 2014 | 6 | - | - | - | - | 17.23 | - | 2.98 |
| 13 August 2014 | 1 | - | - | - | - | 8.37 | - | 4.600 |
| 13 August 2014 | 2 | - | - | - | - | 7.15 | - | 0.639 |
| 13 August 2014 | 3 | - | - | - | - | 12.59 | - | 0.055 |
| 13 August 2014 | 4 | - | - | - | - | 11.07 | - | 0.608 |
| 13 August 2014 | 5 | - | - | - | - | 6.51 | - | 1.67 |
| 13 August 2014 | 6 | - | - | - | - | 19.23 | - | 3.50 |
| 27 August 2014 | 1 | 1.44 | 1.35 | 1.14 | 9.1 | 9.07 | 4.5 | 4.827 |
| 27 August 2014 | 2 | 0.77 | 1.61 | 0.66 | 12.3 | 12.26 | 0.75 | 0.791 |
| 27 August 2014 | 3 | 19.4 | 2.7 | 1.15 | 12.5 | 12.47 | 0.053 | 0.066 |
| 27 August 2014 | 4 | 0.95 | 1.88 | 0.69 | 14.9 | 14.17 | 0.56 | 0.608 |
| 27 August 2014 | 5 | 0.58 | 0.86 | 0.44 | 6.5 | 6.59 | 1.7 | 1.75 |
| 27 August 2014 | 6 | 1.92 | 2.9 | 1.15 | 23 | 23.61 | 3.4 | 3.62 |
| 10 September 2014 | 1 | - | - | - | - | 9.64 | - | 4.923 |
| 10 September 2014 | 2 | - | - | - | - | 12.31 | - | 0.872 |
| 10 September 2014 | 3 | - | - | - | - | 12.53 | - | 0.062 |
| 10 September 2014 | 4 | - | - | - | - | 12.63 | - | 0.562 |
| 10 September 2014 | 5 | - | - | - | - | 6.65 | - | 1.79 |
| 10 September 2014 | 6 | - | - | - | - | 22.76 | - | 3.75 |
| 24 September 2014 | 1 | - | - | - | - | 10.26 | - | 4.891 |
| 24 September 2014 | 2 | - | - | - | - | 17.14 | - | 1.048 |
| 24 September 2014 | 3 | - | - | - | - | 12.70 | - | 0.073 |
| 24 September 2014 | 4 | - | - | - | - | 22.03 | - | 0.557 |
| 24 September 2014 | 5 | - | - | - | - | 6.62 | - | 1.79 |
| 24 September 2014 | 6 | - | - | - | - | 28.77 | - | 3.50 |
| 8 October 2014 | 1 | - | - | - | - | 12.37 | - | 5.157 |
| 8 October 2014 | 2 | - | - | - | - | 17.88 | - | 0.968 |
| 8 October 2014 | 3 | - | - | - | - | 12.96 | - | 0.077 |
| 8 October 2014 | 4 | - | - | - | - | 24.35 | - | 0.623 |
| 8 October 2014 | 5 | - | - | - | - | 6.67 | - | 1.76 |
| 8 October 2014 | 6 | - | - | - | - | 29.98 | - | 3.52 |
| 22 October 2014 | 1 | 2.00 | 1.78 | 1.46 | 12.8 | 12.64 | 5.50 | 5.207 |
| 22 October 2014 | 2 | 1.14 | 2.50 | 1.35 | 18.3 | 18.23 | 1.08 | 1.080 |
| 22 October 2014 | 3 | 19.50 | 2.80 | 1.07 | 13.0 | 12.53 | 0.07 | 0.071 |
| 22 October 2014 | 4 | 1.60 | 3.20 | 1.23 | 26.0 | 22.87 | 0.67 | 0.690 |
| 22 October 2014 | 5 | 0.61 | 0.88 | 0.44 | 7.0 | 6.69 | 1.77 | 1.86 |
| 22 October 2014 | 6 | 2.30 | 3.70 | 1.46 | 32.0 | 30.44 | 3.90 | 3.66 |
| 5 November 2014 | 1 | - | - | - | - | 12.73 | - | 5.294 |
| 5 November 2014 | 2 | - | - | - | - | 18.48 | - | 1.174 |
| 5 November 2014 | 3 | - | - | - | - | 12.59 | - | 0.088 |
| 5 November 2014 | 4 | - | - | - | - | 23.50 | - | 0.676 |
| 5 November 2014 | 5 | - | - | - | - | 6.64 | - | 1.79 |
| 5 November 2014 | 6 | - | - | - | - | 32.06 | - | 3.68 |
| 19 November 2014 | 1 | - | - | - | - | 12.78 | - | 5.267 |
| 19 November 2014 | 2 | - | - | - | - | 18.43 | - | 1.295 |
| 19 November 2014 | 3 | - | - | - | - | 13.19 | - | 0.079 |
| 19 November 2014 | 4 | - | - | - | - | 20.07 | - | 0.642 |
| 19 November 2014 | 5 | - | - | - | - | 6.86 | - | 1.80 |
| 19 November 2014 | 6 | - | - | - | - | 31.51 | - | 3.93 |
| 3 December 2014 | 1 | - | - | - | - | 12.53 | - | 4.943 |
| 3 December 2014 | 2 | - | - | - | - | 16.41 | - | 1.156 |
| 3 December 2014 | 3 | - | - | - | - | 13.15 | - | 0.097 |
| 3 December 2014 | 4 | - | - | - | - | 22.22 | - | 0.729 |
| 3 December 2014 | 5 | - | - | - | - | 6.77 | - | 1.87 |
| 3 December 2014 | 6 | - | - | - | - | 34.01 | - | 4.13 |
| 17 December 2014 | 1 | 2.20 | 1.81 | 1.83 | 12.80 | 12.03 | 5.00 | 5.052 |
| 17 December 2014 | 2 | 1.33 | 2.50 | 1.38 | 16.80 | 16.67 | 1.16 | 1.157 |
| 17 December 2014 | 3 | 19.90 | 2.90 | 1.22 | 13.50 | 13.08 | 0.08 | 0.084 |
| 17 December 2014 | 4 | 1.42 | 2.80 | 1.23 | 21.00 | 19.68 | 0.70 | 0.676 |
| 17 December 2014 | 5 | 0.64 | 0.92 | 0.47 | 7.00 | 6.77 | 1.83 | 1.88 |
| 17 December 2014 | 6 | 2.50 | 4.10 | 1.54 | 33.00 | 33.68 | 4.20 | 4.11 |
| 14 January 2015 | 1 | - | - | - | - | 10.02 | - | 4.839 |
| 14 January 2015 | 2 | - | - | - | - | 8.30 | - | 0.724 |
| 14 January 2015 | 3 | - | - | - | - | 13.35 | - | 0.097 |
| 14 January 2015 | 4 | - | - | - | - | 15.56 | - | 0.742 |
| 14 January 2015 | 5 | - | - | - | - | 6.84 | - | 1.97 |
| 14 January 2015 | 6 | - | - | - | - | 29.69 | - | 4.42 |
| 28 January 2015 | 1 | - | - | - | - | * | - | * |
| 28 January 2015 | 2 | - | - | - | - | 8.38 | - | 0.777 |
| 28 January 2015 | 3 | - | - | - | - | 13.19 | - | 0.097 |
| 28 January 2015 | 4 | - | - | - | - | 14.96 | - | 0.813 |
| 28 January 2015 | 5 | - | - | - | - | 6.93 | - | 1.99 |
| 28 January 2015 | 6 | - | - | - | - | 29.39 | - | 4.46 |
| 11 February 2015 | 1 | - | - | - | - | 11.66 | - | 5.327 |
| 11 February 2015 | 2 | - | - | - | - | 8.33 | - | 0.803 |
| 11 February 2015 | 3 | - | - | - | - | 13.75 | - | 0.085 |
| 11 February 2015 | 4 | - | - | - | - | 16.27 | - | 0.875 |
| 11 February 2015 | 5 | - | - | - | - | 7.15 | - | 2.04 |
| 11 February 2015 | 6 | - | - | - | - | 30.86 | - | 4.58 |
| 2 April 2015 | 1 | - | - | - | - | 14.69 | - | 5.218 |
| 2 April 2015 | 2 | - | - | - | - | 7.21 | - | 0.662 |
| 2 April 2015 | 3 | - | - | - | - | 13.49 | - | 0.109 |
| 2 April 2015 | 4 | - | - | - | - | 15.32 | - | 0.724 |
| 2 April 2015 | 5 | - | - | - | - | 7.10 | - | 2.04 |
| 2 April 2015 | 6 | - | - | - | - | 34.99 | - | 5.40 |
| 1 July 2015 | 1 | - | - | - | - | 6.98 | - | 3.231 |
| 1 July 2015 | 2 | - | - | - | - | 12.12 | - | 0.800 |
| 1 July 2015 | 3 | - | - | - | - | 12.69 | - | 0.146 |
| 1 July 2015 | 4 | - | - | - | - | 11.30 | - | 0.547 |
| 1 July 2015 | 5 | - | - | - | - | 11.35 | - | 0.76 |
| 1 July 2015 | 6 | - | - | - | - | 28.42 | - | 4.74 |

**Table S3.** Results relating to trace elements present at ppm levels. *Interpretation*: Units: all concentrations in µg/L (mg/m^3^) (parts per billion); Al: total aluminium measured by ICP-MS (Inductively Couple Plasma Mass Spectrometry); As: total arsenic measured by ICP-MS; Cd: total cadmium measured by ICP-MS; Cu: total copper measured by ICP-MS; Fe: total iron measured by ICP-MS; Pb: total lead measured by ICP-MS; Pb AAS: acid recoverable lead measured by Atomic Absorption Spectroscopy; Mn: total manganese measured by ICP-MS; “<” sign indicates not detected at concentrations above the figure specified. * Two samples considered to be sampling outliers. **Five low-level measurements for Pb in Tank 3 removed as analytical outliers.

| **Sampling Date** | **Tank** | **Al** | **As** | **Cd** | **Cu** | **Fe** | **Pb** | **Pb AAS** | **Mn** |
| --- | --- | --- | --- | --- | --- | --- | --- | --- | --- |
| 12 February 2014 | 1 | - | - | - | - | - | - | * | - |
| 12 February 2014 | 2 | - | - | - | - | - | - | 26.0 | - |
| 12 February 2014 | 3 | - | - | - | - | - | - | ** | - |
| 12 February 2014 | 4 | - | - | - | - | - | - | 16.2 | - |
| 12 February 2014 | 5 | - | - | - | - | - | - | 7.3 | - |
| 12 February 2014 | 6 | - | - | - | - | - | - | 4.4 | - |
| 26 February 2014 | 1 | - | - | - | - | - | - | 12.3 | - |
| 26 February 2014 | 2 | - | - | - | - | - | - | 26.9 | - |
| 26 February 2014 | 3 | - | - | - | - | - | - | 0.8 | - |
| 26 February 2014 | 4 | - | - | - | - | - | - | 13.3 | - |
| 26 February 2014 | 5 | - | - | - | - | - | - | 9.6 | - |
| 26 February 2014 | 6 | - | - | - | - | - | - | 3.9 | - |
| 12March 2014 | 1 | 6.2 | <1 | <0.05 | 6.1 | <21 | 12.7 | 14.0 | 5.9 |
| 12March 2014 | 2 | 18.0 | <1 | <0.05 | 9.7 | <21 | 39.0 | 41.1 | 17.6 |
| 12March 2014 | 3 | 21.0 | <1 | <0.05 | 23 | <21 | 1.3 | 0.6 | <0.5 |
| 12March 2014 | 4 | 10.7 | <1 | <0.05 | 9.5 | <21 | 16.4 | 13.6 | 4.6 |
| 12March 2014 | 5 | <3.2 | 3.1 | <0.05 | 3.9 | <21 | 6.9 | 7.4 | 1.67 |
| 12March 2014 | 6 | 3.6 | <1 | <0.05 | 3.8 | <21 | 7.0 | 5.6 | 2.5 |
| 26March 2014 | 1 | - | - | - | - | - | - | 12.2 | - |
| 26March 2014 | 2 | - | - | - | - | - | - | 28.5 | - |
| 26March 2014 | 3 | - | - | - | - | - | - | 0.6 | - |
| 26March 2014 | 4 | - | - | - | - | - | - | 19.6 | - |
| 26March 2014 | 5 | - | - | - | - | - | - | 4.0 | - |
| 26March 2014 | 6 | - | - | - | - | - | - | 4.1 | - |
| 9 April 2014 | 1 | - | - | - | - | - | - | 24.4 | - |
| 9 April 2014 | 2 | - | - | - | - | - | - | 32.9 | - |
| 9 April 2014 | 3 | - | - | - | - | - | - | ** | - |
| 9 April 2014 | 4 | - | - | - | - | - | - | 12.5 | - |
| 9 April 2014 | 5 | - | - | - | - | - | - | 6.0 | - |
| 9 April 2014 | 6 | - | - | - | - | - | - | 4.7 | - |
| 23 April 2014 | 1 | - | - | - | - | - | - | 14.5 | - |
| 23 April 2014 | 2 | - | - | - | - | - | - | 24.8 | - |
| 23 April 2014 | 3 | - | - | - | - | - | - | 1.0 | - |
| 23 April 2014 | 4 | - | - | - | - | - | - | 12.9 | - |
| 23 April 2014 | 5 | - | - | - | - | - | - | 6.2 | - |
| 23 April 2014 | 6 | - | - | - | - | - | - | 3.3 | - |
| 7 May 2014 | 1 | 4.9 | <1 | <0.05 | 6.2 | <21 | 10.4 | 9.7 | 5.1 |
| 7 May 2014 | 2 | 10.0 | <1 | <0.05 | 8.6 | <21 | 27.0 | 22.5 | 12.3 |
| 7 May 2014 | 3 | 19.7 | <1 | <0.05 | 25 | <21 | 1.0 | 1.1 | <0.5 |
| 7 May 2014 | 4 | 4.9 | <1 | <0.05 | 13.7 | <21 | 18.1 | 13.4 | 2.4 |
| 7 May 2014 | 5 | <3.2 | 4.0 | 0.06 | 5.5 | <21 | 9.1 | 6.7 | 2.3 |
| 7 May 2014 | 6 | <3.2 | <1 | <0.05 | 4.5 | <21 | 6.4 | 3.0 | 2.0 |
| 21 May 2014 | 1 | - | - | - | - | - | - | 8.3 | - |
| 21 May 2014 | 2 | - | - | - | - | - | - | 21.5 | - |
| 21 May 2014 | 3 | - | - | - | - | - | - | ** | - |
| 21 May 2014 | 4 | - | - | - | - | - | - | 13.0 | - |
| 21 May 2014 | 5 | - | - | - | - | - | - | 7.8 | - |
| 21 May 2014 | 6 | - | - | - | - | - | - | 9.1 | - |
| 4 June 2014 | 1 | - | - | - | - | - | - | 6.3 | - |
| 4 June 2014 | 2 | - | - | - | - | - | - | 22.6 | - |
| 4 June 2014 | 3 | - | - | - | - | - | - | 0.3 | - |
| 4 June 2014 | 4 | - | - | - | - | - | - | 17.4 | - |
| 4 June 2014 | 5 | - | - | - | - | - | - | 7.3 | - |
| 4 June 2014 | 6 | - | - | - | - | - | - | 7.4 | - |
| 18 June 2014 | 1 | - | - | - | - | - | - | 8.9 | - |
| 18 June 2014 | 2 | - | - | - | - | - | - | 24.4 | - |
| 18 June 2014 | 3 | - | - | - | - | - | - | 0.9 | - |
| 18 June 2014 | 4 | - | - | - | - | - | - | 22.8 | - |
| 18 June 2014 | 5 | - | - | - | - | - | - | 11.1 | - |
| 18 June 2014 | 6 | - | - | - | - | - | - | 9.5 | - |
| 2 July 2014 | 1 | 4.6 | <1 | <0.05 | 1.03 | <21 | 9.3 | 22.4 | 5.7 |
| 2 July 2014 | 2 | 8.3 | <1 | <0.05 | 6.41 | <21 | 23.1 | 19.8 | 8.5 |
| 2 July 2014 | 3 | 20.5 | <1 | <0.05 | 21 | <21 | 0.7 | 0.2 | <0.5 |
| 2 July 2014 | 4 | 7.9 | <1 | <0.05 | 2.38 | <21 | 21.2 | 16.9 | 3.2 |
| 2 July 2014 | 5 | 3.6 | 5.3 | 0.12 | 5.54 | <21 | 10.9 | 8.0 | 3.0 |
| 2 July 2014 | 6 | 4.4 | <1 | <0.05 | 1.53 | <21 | 14.0 | 9.2 | 2.8 |
| 16 July 2014 | 1 | - | - | - | - | - | - | 8.0 | - |
| 16 July 2014 | 2 | - | - | - | - | - | - | 20.0 | - |
| 16 July 2014 | 3 | - | - | - | - | - | - | 0.2 | - |
| 16 July 2014 | 4 | - | - | - | - | - | - | 15.8 | - |
| 16 July 2014 | 5 | - | - | - | - | - | - | 8.3 | - |
| 16 July 2014 | 6 | - | - | - | - | - | - | 8.1 | - |
| 30 July 2014 | 1 | - | - | - | - | - | - | 7.7 | - |
| 30 July 2014 | 2 | - | - | - | - | - | - | 17.0 | - |
| 30 July 2014 | 3 | - | - | - | - | - | - | 0.2 | - |
| 30 July 2014 | 4 | - | - | - | - | - | - | 10.6 | - |
| 30 July 2014 | 5 | - | - | - | - | - | - | 11.4 | - |
| 30 July 2014 | 6 | - | - | - | - | - | - | 9.1 | - |
| 13 August 2014 | 1 | - | - | - | - | - | - | 8.1 | - |
| 13 August 2014 | 2 | - | - | - | - | - | - | 14.0 | - |
| 13 August 2014 | 3 | - | - | - | - | - | - | 0.5 | - |
| 13 August 2014 | 4 | - | - | - | - | - | - | 16.2 | - |
| 13 August 2014 | 5 | - | - | - | - | - | - | 9.1 | - |
| 13 August 2014 | 6 | - | - | - | - | - | - | 10.8 | - |
| 27 August 2014 | 1 | 4.9 | <1 | <0.05 | 1.3 | <21 | 8.3 | 7.4 | 4.6 |
| 27 August 2014 | 2 | 5.3 | <1 | <0.05 | 6.5 | <21 | 15.5 | 13.8 | 4.0 |
| 27 August 2014 | 3 | 21 | <1 | <0.05 | 23 | 21 | 2.5 | 2.5 | 0.8 |
| 27 August 2014 | 4 | 6.8 | <1 | <0.05 | 8.6 | <21 | 17.9 | 18.2 | 2.3 |
| 27 August 2014 | 5 | 3.4 | 5.6 | 0.11 | 6.9 | <21 | 11.8 | 8.7 | 3.1 |
| 27 August 2014 | 6 | <3.2 | <1 | <0.05 | 2.4 | <21 | 14.7 | 13.4 | 3.1 |
| 10 September 2014 | 1 | - | - | - | - | - | - | 7.3 | - |
| 10 September 2014 | 2 | - | - | - | - | - | - | 14.8 | - |
| 10 September 2014 | 3 | - | - | - | - | - | - | 0.8 | - |
| 10 September 2014 | 4 | - | - | - | - | - | - | 17.8 | - |
| 10 September 2014 | 5 | - | - | - | - | - | - | 10.5 | - |
| 10 September 2014 | 6 | - | - | - | - | - | - | 11.5 | - |
| 24 September 2014 | 1 | - | - | - | - | - | - | 6.3 | - |
| 24 September 2014 | 2 | - | - | - | - | - | - | 17.0 | - |
| 24 September 2014 | 3 | - | - | - | - | - | - | ** | - |
| 24 September 2014 | 4 | - | - | - | - | - | - | 18.5 | - |
| 24 September 2014 | 5 | - | - | - | - | - | - | 9.2 | - |
| 24 September 2014 | 6 | - | - | - | - | - | - | 11.6 | - |
| 8 October 2014 | 1 | - | - | - | - | - | - | 6.8 | - |
| 8 October 2014 | 2 | - | - | - | - | - | - | 19.5 | - |
| 8 October 2014 | 3 | - | - | - | - | - | - | ** | - |
| 8 October 2014 | 4 | - | - | - | - | - | - | 19.6 | - |
| 8 October 2014 | 5 | - | - | - | - | - | - | 10.6 | - |
| 8 October 2014 | 6 | - | - | - | - | - | - | 11.8 | - |
| 22 October 2014 | 1 | 5.0 | <1 | <0.05 | 2.4 | <21 | 8.7 | 7.0 | 5.8 |
| 22 October 2014 | 2 | 11.5 | <1 | 0.06 | 14.3 | <21 | 31.0 | 26.0 | 7.2 |
| 22 October 2014 | 3 | 19.2 | <1 | <0.05 | 21 | 24 | 0.3 | 1.5 | 0.7 |
| 22 October 2014 | 4 | 8.9 | <1 | <0.05 | 3.4 | <21 | 25.0 | 24.8 | 4.5 |
| 22 October 2014 | 5 | 3.8 | 5.1 | 0.12 | 9.7 | <21 | 13.2 | 11.1 | 3.4 |
| 22 October 2014 | 6 | 7.0 | <1 | 0.07 | 4.5 | <21 | 15.7 | 11.8 | 4.4 |
| 5 November 2014 | 1 | - | - | - | - | - | - | 9.0 | - |
| 5 November 2014 | 2 | - | - | - | - | - | - | 28.6 | - |
| 5 November 2014 | 3 | - | - | - | - | - | - | 0.5 | - |
| 5 November 2014 | 4 | - | - | - | - | - | - | 23.4 | - |
| 5 November 2014 | 5 | - | - | - | - | - | - | 10.4 | - |
| 5 November 2014 | 6 | - | - | - | - | - | - | 12.6 | - |
| 19 November 2014 | 1 | - | - | - | - | - | - | 15.9 | - |
| 19 November 2014 | 2 | - | - | - | - | - | - | 33.4 | - |
| 19 November 2014 | 3 | - | - | - | - | - | - | 0.9 | - |
| 19 November 2014 | 4 | - | - | - | - | - | - | 23.6 | - |
| 19 November 2014 | 5 | - | - | - | - | - | - | 11.6 | - |
| 19 November 2014 | 6 | - | - | - | - | - | - | 11.5 | - |
| 3 December 2014 | 1 | - | - | - | - | - | - | 12.2 | - |
| 3 December 2014 | 2 | - | - | - | - | - | - | 36.0 | - |
| 3 December 2014 | 3 | - | - | - | - | - | - | 1.4 | - |
| 3 December 2014 | 4 | - | - | - | - | - | - | 31.0 | - |
| 3 December 2014 | 5 | - | - | - | - | - | - | 11.7 | - |
| 3 December 2014 | 6 | - | - | - | - | - | - | 14.1 | - |
| 17 December 2014 | 1 | 4.1 | <1 | 0.06 | 2.4 | <21 | 9.9 | 11.2 | 6.4 |
| 17 December 2014 | 2 | 19.4 | <1 | 0.07 | 28 | <21 | 39.0 | 32.9 | 9.0 |
| 17 December 2014 | 3 | 17.4 | <1 | <0.05 | 22 | <21 | 0.5 | 1.4 | 0.6 |
| 17 December 2014 | 4 | 8.0 | <1 | <0.05 | 5.9 | <21 | 27.0 | 23.0 | 5.5 |
| 17 December 2014 | 5 | 4.6 | 5.7 | 0.14 | 11.2 | <21 | 16.3 | 12.7 | 3.6 |
| 17 December 2014 | 6 | 10.2 | <1 | 0.06 | 3.1 | <21 | 16.4 | 12.6 | 4.6 |
| 14 January 2015 | 1 | - | - | - | - | - | - | 10.5 | - |
| 14 January 2015 | 2 | - | - | - | - | - | - | 22.6 | - |
| 14 January 2015 | 3 | - | - | - | - | - | - | 0.3 | - |
| 14 January 2015 | 4 | - | - | - | - | - | - | 33.4 | - |
| 14 January 2015 | 5 | - | - | - | - | - | - | 14.8 | - |
| 14 January 2015 | 6 | - | - | - | - | - | - | 13.9 | - |
| 28 January 2015 | 1 | - | - | - | - | - | - | * | - |
| 28 January 2015 | 2 | - | - | - | - | - | - | 25.3 | - |
| 28 January 2015 | 3 | - | - | - | - | - | - | 0.6 | - |
| 28 January 2015 | 4 | - | - | - | - | - | - | 38.0 | - |
| 28 January 2015 | 5 | - | - | - | - | - | - | 14.5 | - |
| 28 January 2015 | 6 | - | - | - | - | - | - | 16.3 | - |
| 11 February 2015 | 1 | - | - | - | - | - | - | 8.4 | - |
| 11 February 2015 | 2 | - | - | - | - | - | - | 22.8 | - |
| 11 February 2015 | 3 | - | - | - | - | - | - | 0.5 | - |
| 11 February 2015 | 4 | - | - | - | - | - | - | 38.0 | - |
| 11 February 2015 | 5 | - | - | - | - | - | - | 15.8 | - |
| 11 February 2015 | 6 | - | - | - | - | - | - | 15.1 | - |
| 2 April 2015 | 1 | - | - | - | - | - | - | 6.5 | - |
| 2 April 2015 | 2 | - | - | - | - | - | - | 8.5 | - |
| 2 April 2015 | 3 | - | - | - | - | - | - | 0.1 | - |
| 2 April 2015 | 4 | - | - | - | - | - | - | 12.8 | - |
| 2 April 2015 | 5 | - | - | - | - | - | - | 13.2 | - |
| 2 April 2015 | 6 | - | - | - | - | - | - | 9.4 | - |
| 1 July 2015 | 1 | - | - | - | - | - | - | 6.7 | - |
| 1 July 2015 | 2 | - | - | - | - | - | - | 10.8 | - |
| 1 July 2015 | 3 | - | - | - | - | - | - | 0.3 | - |
| 1 July 2015 | 4 | - | - | - | - | - | - | 12.6 | - |
| 1 July 2015 | 5 | - | - | - | - | - | - | 7.6 | - |
| 1 July 2015 | 6 | - | - | - | - | - | - | 13.6 | - |

**Table S4.** Identities and detection limits of organic compounds tested by gas chromatography-mass spectrometry (GC-MS). *Notes:* Samples collected from all six tanks on 11 February 2015 were tested for 79 trace organic compounds by GC-MS. None of the target compounds were detected in any of the six tanks. The identities of the compounds tested for and their reported detection limits (parts per million) are provided in Table S4.

| **Compound Name(s)** | **Detection Limit (mg/L)** | **Compound Name(s)** | **Detection Limit (mg/L)** |
| --- | --- | --- | --- |
| Benzene | <0.0010 | 3,3’-Dichlorobenzidine | <0.003 |
| Toluene | <0.0010 | 2,4-Dichlorophenol | <0.0005 |
| Ethylbenzene | <0.0010 | Dieldrin | <0.0005 |
| m&p-Xylene | <0.002 | Diethylphthalate | <0.0010 |
| o-Xylene | <0.0010 | 2,4-Dimethylphenol | <0.0005 |
| Acenaphthene | <0.0003 | Dimethylphthalate | <0.0010 |
| Acenaphthylene | <0.0003 | Di-n-butylphthalate | <0.0010 |
| Aldrin | <0.0005 | 2,4-Dinitrotoluene | <0.0010 |
| Anthracene | <0.0003 | 2,6-Dinitrotoluene | <0.0010 |
| Benzo[a]anthracene | <0.0003 | Di-n-octylphthalate | <0.0010 |
| Benzo[a]pyrene (BAP) | <0.0005 | Endosulfan I | <0.0010 |
| Benzo[b]fluoranthene + Benzo[j]fluoranthene | <0.0005 | Endosulfan II | <0.0010 |
| Benzo[g,h,i]perylene | <0.0005 | Endosulfan sulfate | <0.0010 |
| Benzo[k]fluoranthene | <0.0005 | Endrin | <0.0010 |
| Benzyl alcohol | <0.005 | Endrin ketone | <0.0010 |
| alpha-BHC | <0.0005 | Fluoranthene | <0.0003 |
| beta-BHC | <0.0005 | Fluorene | <0.0003 |
| delta-BHC | <0.0005 | Heptachlor | <0.0005 |
| gamma-BHC (Lindane) | <0.0005 | Heptachlor epoxide | <0.0005 |
| Bis(2-chloroethoxy) methane | <0.0005 | Hexachlorobenzene | <0.0005 |
| Bis(2-chloroethyl)ether | <0.0005 | Hexachlorobutadiene | <0.0010 |
| Bis(2-chloroisopropyl)ether | <0.0005 | Hexachloroethane | <0.0010 |
| Bis(2-ethylhexyl)phthalate | <0.003 | Indeno(1,2,3-c,d)pyrene | <0.0005 |
| 4-Bromophenyl phenyl ether | <0.0005 | Isophorone | <0.0005 |
| Butylbenzylphthalate | <0.0010 | 2-Methylnaphthalene | <0.0003 |
| Carbazole | <0.0005 | 3 & 4-Methylphenol (m- + p-cresol) | <0.0010 |
| 4-Chloro-3-methylphenol | <0.0010 | 2-Methylphenol (o-Cresol) | <0.0005 |
| 2-Chloronaphthalene | <0.0003 | Naphthalene | <0.0003 |
| 2-Chlorophenol | <0.0005 | Nitrobenzene | <0.0005 |
| 4-Chlorophenyl phenyl ether | <0.0005 | 2-Nitrophenol | <0.0010 |
| Chrysene | <0.0003 | N-Nitrosodi-n-propylamine | <0.0010 |
| 4,4’-DDD | <0.0005 | N-Nitrosodiphenylamine | <0.0010 |
| 4,4’-DDE | <0.0005 | Pentachlorophenol (PCP) | <0.010 |
| 4,4’-DDT | <0.0010 | Phenanthrene | <0.0003 |
| Di(2-ethylhexyl)adipate | <0.0010 | Phenol | <0.0010 |
| Dibenzo[a,h]anthracene | <0.0005 | Pyrene | <0.0003 |
| Dibenzofuran | <0.0005 | 1,2,4-Trichlorobenzene | <0.0005 |
| 1,2-Dichlorobenzene | <0.0010 | 2,4,5-Trichlorophenol | <0.0010 |
| 1,3-Dichlorobenzene | <0.0010 | 2,4,6-Trichlorophenol | <0.0010 |
| 1,4-Dichlorobenzene | <0.0010 | - | - |

**Table S5.** Identities and detection limits of organic compounds tested by GC-MS. *Notes:* During this project, additional samples were collected of rainwater (5 November 2015), and water from a 400 L roof-water storage tank (Tank 7) draining a polycarbonate roof, located in Rose St, Porirua (26 February and 30 June 2015). The 400-L tank was re-sampled as preliminary results showed a very low pH. Results for tested variables are provided below.

| **Variable** | **Units** | **Rainwater** | **Tank 7** | |
| --- | --- | --- | --- | --- |
|  |  | **5 November 2014** | **26 February 2014** | **30 June 15** |
| pH | pH units | 5.19 | 3.25 | 3.19 |
| Conductivity | µS/cm | 32.3 | 249.5 | 244.0 |
| Total dissolved solids | mg/L | 16.1 | 124.5 | 122.0 |
| Turbidity | NTU | 0.97 | 0.41 | 0.27 |
| Ca | mg/L | 0.21 | - | - |
| Mg | mg/L | 0.53 | - | - |
| K | mg/L | 0.21 | - | - |
| Na | mg/L | 3.9 | - | - |
| Na AAS | mg/L | 4.02 | 19.4 | 18.95 |
| Zn | mg/L | 0.027 | - | - |
| Zn AAS | mg/L | - | 0.18 | 0.143 |
| Pb AAS | mg/L | - | 0.0262 | 0.0249 |
| Mn | mg/L | 0.00064 | - | - |
| Al | mg/L | 0.0086 | - | - |
| As | mg/L | <0.0011 | - | - |
| Cd | mg/L | <0.000053 | - | - |
| Cu | mg/L | 0.00131 | - | - |
| Fe | mg/L | <0.021 | - | - |
| Pb | mg/L | 0.00039 | - | - |
| Pb AAS | mg/L | - | 0.0262 | 0.0249 |
| Mn | mg/L | 0.00064 | - | - |

2. Further Detail on Major Cation Composition of Tank Waters

**Table S6.** Reconciliation of measured conductivities with major cation concentrations.

| **Quantity** | | **Tank 1** | **Tank 2** | **Tank 4** | **Tank 5** | **Tank 6** | **All Rain-Fed Tanks** | **Tank 3 (Tap Water Control)** |
| --- | --- | --- | --- | --- | --- | --- | --- | --- |
| Mean measured Conductivity (µS/cm) | | 97.2 | 106.2 | 120.5 | 53.2 | 169.9 | - | 187.4 |
| Estimated Percent (%) Contributions to Measured Conductivity Assuming Counter-Ions Specified | From Na^+^ as NaCl | 58.6 | 66.5 | 72.9 | 62.6 | 66.2 | 70.6 | 37.8 |
|  | From K^+^ as KCl | 5.6 | 3.9 | 2.6 | 2.9 | 3.5 | 2.3 | 2.4 |
|  | From Ca^2+^ as CaCl_2_ | 10.4 | 6.2 | 6.0 | 6.5 | 7.0 | 5.9 | 64.1 |
|  | From Mg^2+^ as MgCl_2_ | 13.4 | 14.8 | 14.6 | 12.6 | 13.8 | 13.5 | 12.8 |
|  | From Zn^2+^ as ZnSO_4_ | 7.2 | 1.3 | 0.8 | 4.3 | 3.3 | 3.1 | 0.0 |
|  | From H^+^ as Acetic Acid | 0.1 | 6.4 | 1.2 | 2.8 | 2.1 | 0.2 | 0.0 |
|  | Sum of Above | 95.3 | 99.0 | 98.0 | 91.7 | 95.9 | 95.5 | 117.0 |

3. Source Apportionment Calculations

A simple source-apportionment approach was used to estimate the potential influence of sea-salt aerosol to total sodium on average and during a period when conductivity and total sodium showed their largest increases. This took the form of estimating the best fit to observed ratios of Na/K, Na/Ca, and Na/Mg in each tank based on the assumption that these were derived from combinations of (a) bulk sea-salt aerosol with cation ratios Na/K = 29.6, Na/Ca = 22.87, and Na/Mg = 8.81 [1], and (b) ratios characteristic of soil or crustal material taken to approximate other possible sources Na/K = 0.71, Na/Ca = 0.33, and Na/Mg = 0.59 [2]. Estimates of average (and peak in brackets) contributions made by sea-salt aerosol to sodium and conductivity in rain-fed tanks are as follows: Tank 1, 26% (34%); Tank 2, 49% (50%); Tank 4, 66% (72%); Tank 5, 51% (73%); Tank 6 69% (100%). Differences between tanks are likely to reflect differences in proximity to the sea, location in relation to the dominant wind-directions, height, microclimate, roof-surface direction, seasonality of sea-salt influence, and how inputs are counteracted by dilution in heavy rainfall. Although all properties were within the same medium risk corrosion zone (Section 2.3), the influence of proximity to the sea was evident with a modest (*p* < 0.01) negative correlation between distance to south coast and sodium concentrations in the rain-fed tanks. Over the first year, sodium concentrations in Tank 6 increased by ~2.2 mg/L per month. Assuming sea-salt aerosol was the dominant sodium source, this would be equivalent to entry of about 40 mL of seawater per month.

Reference

1. Keene, W.C.; Maring, H.; Maben, J.R.; Kieber, D.J.; Pszenny, A.A.; Dahl, E.E.; Izaguirre, M.A.; Davis, A.J.; Long, M.S.; Zhou, X.; et al. Chemical and physical characteristics of nascent aerosols produced by bursting bubbles at a model air-sea interface. *J. Geophys. Res.* **2007**, *112*. doi:10.1029/2007JD008464
2. Lide, D.R. *CRC Handbook of Chemistry and Physics*, 85th ed.; CRC (Chemical Rubber Company) Press:
   Boca Raton, FL, USA, 2004.

© 2016 by the authors; licensee MDPI, Basel, Switzerland. This article is an open access article distributed under the terms and conditions of the Creative Commons by Attribution (CC-BY) license (http://creativecommons.org/licenses/by/4.0/).
